# Investigating geographic differences in environmental chemical exposures in maternal and cord sera using non-targeted screening and silicone wristbands in California

**DOI:** 10.1038/s41370-022-00426-9

**Published:** 2022-04-21

**Authors:** Dana E. Goin, Dimitri Abrahamsson, Miaomiao Wang, June-Soo Park, Marina Sirota, Rachel Morello-Frosch, Erin DeMicco, Jessica Trowbridge, Laura August, Steven O’Connell, Subhashini Ladella, Marya G. Zlatnik, Tracey J. Woodruff

**Affiliations:** 1 Program on Reproductive Health and the Environment, Department of Obstetrics, Gynecology, and Reproductive Sciences, University of California San Francisco School of Medicine, San Francisco, CA USA; 2 Environmental Chemistry Laboratory, Department of Toxic Substances Control, California Environmental Protection Agency, Berkeley, CA USA; 3 Bakar Computational Health Sciences Institute and Department of Pediatrics, University of California San Francisco, San Francisco, CA USA; 4 Department of Environmental Science, Policy and Management and School of Public Health, University of California Berkeley, Berkeley, CA USA; 5 Office of Environmental Health Hazard Assessment, California Environmental Protection Agency, Sacramento, CA USA; 6 MyExposome, Inc., Corvallis, OR USA; 7 Fresno Medical Education Program, Department of Obstetrics, Gynecology, and Reproductive Sciences, University of California San Francisco School of Medicine, Fresno, CA USA

**Keywords:** Non-targeted chemical analysis, Silicone wristbands, Exposure assessment, Pregnancy, Pesticide;, Phthalate, Environmental disparities, cord blood

## Abstract

**Background:**

Differential risks for adverse pregnancy outcomes may be influenced by prenatal chemical exposures, but current exposure methods may not fully capture data to identify harms and differences.

**Methods:**

We collected maternal and cord sera from pregnant people in Fresno and San Francisco, and screened for over 2420 chemicals using LC-QTOF/MS. We matched San Francisco participants to Fresno participants (*N* = 150) and compared detection frequencies. Twenty-six Fresno participants wore silicone wristbands evaluated for over 1500 chemicals using quantitative chemical analysis. We assessed whether living in tracts with higher levels of pollution according to CalEnviroScreen correlated with higher numbers of chemicals detected in sera.

**Results:**

We detected 2167 suspect chemical features across maternal and cord sera. The number of suspect chemical features was not different by city, but a higher number of suspect chemicals in cosmetics or fragrances was detected in the Fresno versus San Francisco participants’ sera. We also found high levels of chemicals used in fragrances measured in the silicone wristbands. Fresno participants living in tracts with higher pesticide scores had higher numbers of suspect pesticides in their sera.

**Conclusions:**

Multiple exposure-assessment approaches can identify exposure to many chemicals during pregnancy that have not been well-studied for health effects.

## Background

Exposure to chemicals during pregnancy can influence maternal and fetal health and have lasting impacts on child development [1–3]. While we know that women in the United States (US) are exposed to many chemicals during pregnancy [3, 4], the majority of chemicals used in the US have never been evaluated in terms of their burden of exposure among pregnant women nor impacts on maternal and fetal health. Accordingly, there are currently over 86,000 chemicals in the inventories of the Environmental Protection Agency (EPA) [5], half of which are actively in use in the US [6], but fewer than 350 have been included in routine biomonitoring research of pregnant women [4]. Research focusing on the exposome, or the cumulative chemical and non-chemical exposures people experience that affect health, can better represent the true scope of environmental exposures women experience during pregnancy and has potential to better explain disparities in maternal and infant health [7, 8].

California has the largest economy in the US [9], with highly varied industries and occupations across the state. It also is home to one of the most racially and ethnically diverse populations in the country [10]. Therefore, comparison of chemical exposures across regions may help shed light on differential patterns of chemical exposures by geography or structural factors that may play a role in well-documented disparities in adverse maternal and infant health outcomes by race/ethnicity and socioeconomic position within the state. For instance, the region surrounding Fresno has consistently high prevalence of preterm birth compared to the rest of California, and studies have documented higher burden of pollutants, chemical exposures, and social and structural factors present in Fresno that may contribute to this elevated risk [11]. However, a broad screening of multiple chemical exposures among pregnant women and their children comparing levels in the Fresno region to others in California has not previously been done.

New technologies and data are becoming more widely available that can improve measurement of many environmental hazards at multiple levels of exposure. For example, recent advances in non-targeted high resolution mass spectrometry (HRMS) methods provide an opportunity to screen biological samples for a much larger set of chemicals than is currently possible with targeted biomonitoring [12–14]. We have previously used HRMS to identify novel chemical exposures during pregnancy [15]; the HRMS methods have also been used for studies of the exposome by EPA [16] and others [7]. Similarly, silicone wristbands are a relatively novel approach for passive sampling of a multitude of individual exposures in a noninvasive way, which have been used to capture exposure to organophosphate flame retardants, polybrominated diphenyl ethers (PBDEs), and polycyclic aromatic hydrocarbons (PAHs) [17–19]. Furthermore, geographic measures of exposure like California’s efforts to improve mapping and surveillance of environmental hazards through CalEnviroScreen can add further context to how and why exposure to pollutants and other environmental chemicals might vary by geography [20].

To better characterize exposure differentials across California and to compare utility of different chemical exposure-assessment methods, we collected a comprehensive set of exposure data using individual-level serum biomonitoring, wristband passive sampling, and area-based estimates of pollution exposures to compare prenatal exposures to industrial chemicals and pollutants in Fresno versus the San Francisco Bay Area. We collected maternal and cord sera from pregnant women and their newborns at delivery from hospitals in both areas and applied non-targeted suspect screening methods; we used silicone bracelets to passively sample chemicals in Fresno participants to compare to non-targeted chemical identification; and we geocoded participants and linked their Census tracts of residence to CalEnviroScreen, an online spatial tool developed by the California EPA’s Office of Environmental Health Hazard Assessment to identify communities with a high burden of chemical and non-chemical exposures [21].

## Data and methods

### Study population and sample collection

Our Fresno Biomonitoring Study enrolled 78 pregnant women at delivery from Fresno Community Regional Medical Center, which serves a primarily low-income population, with over 89% of births in 2017 covered by Medi-Cal [22], between August and December 2018. Eligibility criteria included patients over the age of 18 and English or Spanish speakers. After informed consent, we administered a survey, collected biospecimens for 75 maternal and 64 cord sera samples at delivery, and linked maternal survey data and medical records for maternal and neonatal biospecimen samples.

San Francisco participants were part of our Chemicals in Our Bodies-2 (CIOB2) study recruited from UCSF Mission Bay, UCSF Moffit Long, and Zuckerberg San Francisco General Hospitals during their second trimester. We administered questionnaires during this initial visit, and linked the information to medical records. The UCSF Mission Bay and Moffit Long Hospitals serve an economically and racially diverse population, although most have private health insurance. Zuckerberg San Francisco General Hospital serves a primarily low-income and Latinx population, the majority of whom have public health insurance.

The Fresno Biomonitoring Study and the Chemicals in Our Bodies-2 study protocols were approved by the Institutional Review Boards of the University of California, San Francisco, University of California, Berkeley, and Fresno Community Regional Medical Center (IRB number 17-23688).

**Fig. 1:**
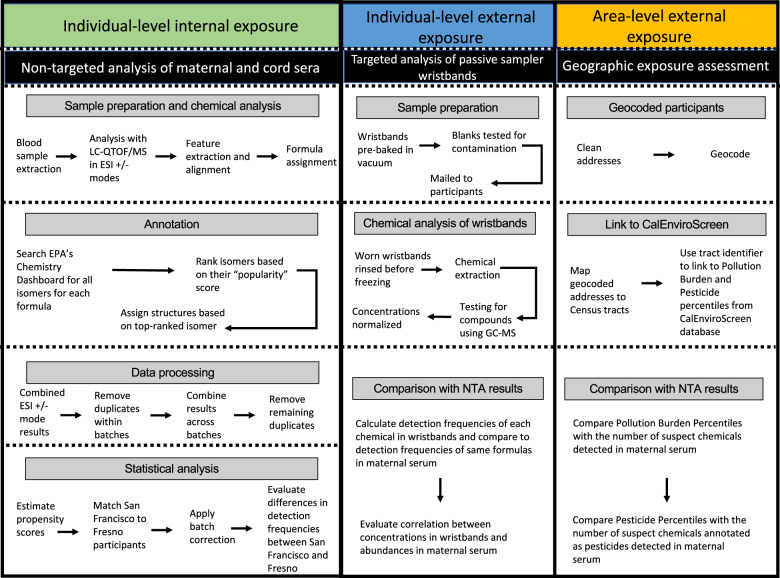
Workflow diagram of individual and area-level exposure-assessment approaches to characterize environmental chemical exposures among pregnant women in Fresno and San Francisco. (1) Workflow for the non-targeted analysis of maternal and cord sera to assess individual-level internal chemical exposures. (2) Workflow for the targeted analysis in the silicone wristbands to assess individual-level external chemical exposures. (3) Workflow for the geographic exposure assessment using CalEnviroScreen to assess area-level external chemical exposures.

An overview of the methods for the non-targeted analysis of sera, targeted analysis of wristbands, and geographic exposure assessment is provided in a workflow diagram (Fig. 1). Additional details are provided below.

### Non-targeted analysis

We conducted a non-targeted HRMS approach to evaluate the chemicals present in maternal and cord sera. This involved chemical analysis, data processing, statistical analysis, and chemical annotation, each of which we describe in detail below. We use the term “suspect chemicals” to refer to chemical features identified as part of this analysis, which reflects the fact that many of the features were not confirmed with analytical standards or matched to MS/MS spectral libraries.

#### Chemical analysis

Maternal and cord blood samples were stored at −80 °C in freezers at the University of California, San Francisco and subsequently transported to the Department of Toxic Substances Control in Berkeley, California on dry ice, where the Environmental Chemistry Laboratory extracted the maternal and neonatal serum by protein precipitation and centrifuging aliquots of 250 μL per sample were spiked with 25 μL 100 ng/L surrogate standard mixture during the extraction process. Isotope labeled standards were used as surrogate, among which M2PFOA was monitored in the negative mode, and D15-Triphenyl Phosphate and DL-Cotinine were used in the positive mode. Additional QA/QC procedures are available in the Supplementary Material and were described in detail previously [23]. The samples were mixed and stored at 4 °C until liquid chromatography quadrupole time-of-flight/mass spectrometry (LC-QTOF/MS) was conducted, using an Agilent UPLC coupled to an Agilent 6550 QTOF (Agilent Technologies, Santa Clara, CA) in both positive and negative electrospray ionization modes. Full scan MS^1^ mass spectra were acquired in the range of 100–1000 Da with resolving power of 40,000 and a mass accuracy of <5 ppm. The QTOF was calibrated and the mass accuracy was corrected with reference standards of reference masses 112.985587 and 1033.988109 for negative mode, and 121.050873 and 922.009798 for positive mode continuously during the run. The UPLC was operated with an Agilent ZORBAX Eclipse Plus C18 column (2.1 × 100 mm, 1.8 μm) and a gradient solvent program of flow rate 0.3 mL/min with 5 mM ammonium acetate in 90% methanol/water increasing the organic phase from 10% to 100% over 15 min, following a 4 min equilibration at 100%.

We included water blanks, matrix blanks, and matrix spikes in each set of 20 sample preparation, and every batch analyzed using LC-QTOF/MS included these blanks. The water blank was used as procedural blank, and only features that were two times or higher in samples were retained. Spiked matrix was used as QC material. Seven OPFRs and ten PFASs were spiked into blank serum and analyzed alongside the samples. The spiked standards were monitored between the batches for consistency. Further description of the matrix blanks is available in the Supplementary Material and has been previously published [23]. We used Agilent MassHunter Profinder to process data and extract features from the raw total ion chromatograms (TIC). Agilent Mass Profiler Professional was used to align the features, and any features found in blanks were subsequently filtered out from the rest of the samples if the intensities in samples were less than twofold those detected in water blanks. The features were matched to formulas using a screening approach with a database of 2420 unique formulas and their isomers. The construction of this database is described in detail elsewhere [24]. The detected chemical features were initially matched to molecular formulas in the database based on mass accuracy, isotopic abundance and patterns with a score threshold of 70.

#### Annotation

We annotated chemical structures with isomers in our database using their most likely isomer. We ran the formulas through an algorithm which creates a score to indicate the most probable isomer [23]. This score is based on the average of the blind probability, i.e., 1 over the total number of isomers for that formula, and a score that combines the number of times an isomer is mentioned in pubmed, pubchem, and the production volume, which were all scaled between 0 and 1. The average of these metrics combines the available information to create a best guess for identification of the isomer. If there were multiple isomers per formula that were differentially detected across cities, we ranked the chemicals according to their detection frequency and then assigned annotations to the isomers, that is, the most probable isomer was matched to the isomer with the highest detection frequency, the second most probable isomer was matched to the isomer with the second highest detection frequency, etc.

#### Data processing

We combined the suspect chemicals detected in negative and positive ionization modes within batches. To do so, we first ranked chemicals within the same formula group based on their detection frequency. We classified chemicals as detected if their abundances were ≥5000. We then compared chemicals within formula groups and identified as duplicates any chemicals that had an experimental mass of within 15 ppm and a retention time of within 0.5 min of one another [25]. For chemicals that had one or more duplicates as determined by these criteria, we retained the one with the highest detection frequency. This resulted in the removal of 134 chemicals from the first batch of San Francisco samples, 112 chemicals from the second batch of San Francisco samples, and 55 chemicals from the Fresno batch of samples. We then combined all three batches. In order to uniquely identify chemicals within batch but allow for matches across batches, we replicated the matching procedure described above in regard to mass and retention time. Additionally, any chemicals that had a match in common, but were not matches themselves, were also considered duplicates. We also removed chemicals that were not previously identified as duplicates within batches. Once the duplication removal process was complete, we averaged the abundances across the two technical replicates. The replicates were manually evaluated to examine the overlay of TIC and the features that were extracted only once in one sample were filtered.

#### Statistical analyses

Given the demographic differences between the Fresno and San Francisco participants we matched women living in the Fresno area to women in the San Francisco Bay Area on maternal age, race/ethnicity, educational attainment, and marital status using propensity scores to better capture chemical exposure differences likely to be attributable to geography rather than sociodemographic factors. We used 1:1 nearest neighbor matching without replacement using propensity scores using the MatchIt R package [26], in which the propensity score represented the probability that a participant lived in Fresno. Supplementary Figs. 1, 2, 3 illustrate the propensity scores before and after matching for both maternal and neonatal samples. We conducted the matching procedure separately for each matrix, although we used the same maternal covariates for both, due to differences in number of maternal and cord sera available. We replicated the matching procedure three times and compared results across iterations to ensure robustness.

Because the samples were analyzed in three separate batches, we used the ComBat R package [27] to remove batch effects after restricting to the matched sample and adjusting for the same confounders used in the propensity score estimation. We log transformed the abundances before applying the batch effect correction to preserve the 0 boundary, and any abundances that were 0 were corrected to 0.1 before log-transformation. We used principal components analysis to assess whether the batch effects were sufficiently removed. The San Francisco samples were analyzed in two separate batches while the Fresno samples were analyzed in their own batch. This complicates removal of batch effects, because it is not clear if the differences between the San Francisco and Fresno samples are due to biological differences or batch effects. We proceeded with batch effect removal with the understanding that this may reduce potentially meaningful differences in chemical detection frequencies between the two regions; therefore, our findings can be interpreted as conservative.

After batch correction, we applied the same detection limit of abundances ≥5000 to all chemicals and used Fisher’s exact test to compare the detection frequencies for each chemical between regions. We used Pearson’s correlation coefficient to evaluate the correlation between log abundances in maternal and cord sera.

### Wristband analysis

We compared the non-targeted chemical analyses results in maternal sera with chemicals detected using silicone wristbands worn by 26 Fresno participants developed for personal environmental monitoring [24] and analyzed by MyExposome, Inc. The wristbands were tested for 1528 potential compounds that women may have been exposed to from atmospheric sources or through direct contact such as sweat on the skin (full list is available at https://www.myexposome.com/fullscreen). Each chemical was categorized into one or more common exposure sources or chemical classes, which include pesticides, pharmaceuticals, chemicals in commerce, personal care products, polycyclic aromatic hydrocarbons (PAHs), volatile organic compounds, flame retardants (PBDEs and PBBs), PCBs, and consumer products based on chemical structure and source data from the hazardous substances database from the national library of medicine (https://pubchem.ncbi.nlm.nih.gov/). To mitigate contamination, MyExposome pre-bakes the silicone wristbands in a vacuum as described elsewhere [28] and tests each batch of wristbands to ensure data quality objectives are met. Wristbands that pass conditioning processes are packaged in airtight Teflon bags, and blank wristbands are tested for chemicals that will be analyzed to ensure the integrity of deployed wristband data. Participants wore the wristbands for an average of 35 days after delivery, were collected by our Fresno team, and then shipped to MyExposome. Wristbands were immediately placed in standard freezers (approx. −20 °C), then rinsed with water filtered through a Barnstead D7389 purifier (Dubuque, IA) and isopropyl alcohol (purity ≥ 99.9)) to remove any surface particulates of the wristband before being placed back in the freezer. Chemicals were extracted using two rounds of ethyl-acetate (purity ≥ 99.9%) and the extract was concentrated down to 1 mL vial using filtered nitrogen. The extract was cleaned further to remove skin-surface oils using solid-phase extraction with C18 cartridges (Supelco, Bellefonte, PA) described elsewhere [29], and then the sample was tested for compounds using gas chromatography–mass spectrometry using a DB-5MS column (Agilent) at an electron impact mode of 70 eV with retention time locking automatic mass spectral deconvolution and identification software (AMDIS) [30]. The concentrations were calculated and normalized by the amount of time the wristbands were worn and the size of the wristband (to control for the amount of silicone). Therefore, the concentrations are reported in terms of nanograms per gram of silicone per week of exposure (i.e., ng/g/week).

The chemicals in the wristbands were detected using AMDIS deconvolution software and spectral libraries to create targeted analyses [31], while the non-targeted analyses only permitted identification of formulas that were annotated using the methods described above. Therefore, to compare the detection of chemicals across the wristband and non-targeted approaches, we only examined the overlap between chemicals that were present in both databases and matched formulas from the non-targeted analyses to those detected in the wristbands. Chemicals with multiple formulas were ranked according to their detection frequency in both sources and matched using formula and rank. To evaluate the relationship between the abundances and ng/g silicone per week for each chemical among individual participants, we limited comparisons to unique formulas across the wristbands and non-targeted analyses.

### CalEnviroScreen

We geocoded participants and identified their Census tracts of residence, which we then linked to geographic measures of pollutant exposure from the draft CalEnviroScreen (CES) 4.0 tool. Details about how those measures are created have been described previously [20]. The tool characterizes Census tracts across California in terms of their exposure and vulnerability to environmental hazards [32]. We used the Pollution Burden percentiles, which scales the raw Pollution Burden scores to provide a relative score to compare with other tracts statewide. The raw Pollution Burden scores are a weighted average of exposure percentiles of ozone and PM2.5 concentrations, diesel PM emissions, drinking water contaminants, pesticide use, toxic releases from facilities, and traffic density with scores representing the proximity to cleanup sites, impaired water bodies, groundwater threats, hazardous waste facilities and generators, and solid waste sites and facilities [33]. To evaluate the relationship between non-targeted exposures and geographically-based measures of environmental hazards, we also used the Pesticide percentiles, which are a scaled version of the Pesticide Scores, representing the total pounds of active pesticide ingredients used in production agriculture per square mile [33]. We compared the Pollution Burden percentiles to the abundances and detection frequencies of chemicals detected in maternal and neonatal serum. We also compared the Pesticide percentiles to the detection frequencies and abundances of likely pesticides in maternal and neonatal serum. To evaluate whether there was an association between the CalEnviroScreen percentiles and number of suspect chemicals detected, we calculated Spearman’s rank correlation coefficients.

## Results

In Fresno, participants were more likely to be younger than 30, to have educational attainment lower than a bachelor’s degree, and to be Latina compared to participants in San Francisco (Supplementary Table 1). After propensity score matching, San Francisco participants were more similar to their Fresno counterparts, although they were still older on average (15% were 35 years or older compared to 4% in Fresno). These patterns were similar for those who had cord serum analyzed (Supplementary Table 1). Fresno participants who wore the wristbands were slightly more likely to have a high school degree, be white, and never married compared to all Fresno participants included in the NTA analysis (Supplementary Table 2).

### Serum NTA results

**Fig. 2:**
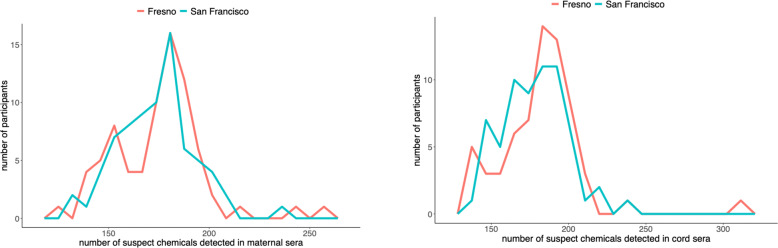
Number of suspect chemicals identified in maternal and cord serum samples by city of residence. Note: Suspect chemicals were considered to be detected if they had abundances exceeding 5000. There were *N* = 75 maternal participants in Fresno and *N* = 75 maternal participants in San Francisco in this analysis, and *N* = 64 cord samples in both cities.

Overall, 2167 features were identified across the maternal and neonatal samples in Fresno and San Francisco (results available at github.com/degoin/Fresno-NTA-exposure-assessment-data). We found participants in Fresno (*N* = 75) had similar numbers of suspect chemicals detected in maternal serum compared to matched San Francisco participants (*N* = 75) (Fig. 2). The mean number of suspect chemicals detected in Fresno participants was 175 (IQR = 160, 186), whereas the mean in San Francisco was 174 (IQR = 161, 184) (*t* test *p* value = 0.64). The log average abundances in maternal serum were correlated between cities (Pearson correlation coefficient = 0.71), but San Francisco had higher log average abundances (*t* test *p* value < 0.001) (Supplementary Fig. 4). These patterns were similar for cord serum (Fig. 2), where the mean number of suspect chemicals detected in Fresno was 182 (IQR = 169, 194) and the mean in San Francisco was 178 (IQR = 163, 193) (*t* test *p* value = 0.27). The log average abundances in cord serum were similarly correlated between cities (Pearson correlation coefficient = 0.71), and San Francisco had higher log average abundances in cord serum (*t* test *p* value < 0.001) (Supplementary Fig. 5). The natural log abundances of chemicals in paired maternal and cord sera were correlated (Pearson correlation coefficient = 0.74).

Among Fresno participants, there were 72 suspect chemicals detected in >90% of maternal samples and 72 suspect chemicals detected in >90% of neonatal samples (56 suspect chemicals were detected in >90% of both maternal and neonatal samples). Among San Francisco participants, 85 suspect chemicals were detected in >90% of maternal samples and 90 in >90% of neonatal samples, with 71 detected in >90% of both maternal and neonatal samples. Next, we assessed the overlap in suspect chemicals detected in both San Francisco and Fresno. In maternal samples, 64 suspect chemicals were detected in at least 90% of maternal samples from both Fresno and San Francisco regions. Similarly, in neonatal samples, 64 suspect chemicals were detected in at least 90% of samples in both cities. Of these frequently detected suspect chemicals, 49 were detected in >90% of both maternal and fetal samples in both cities.

**Fig. 3:**
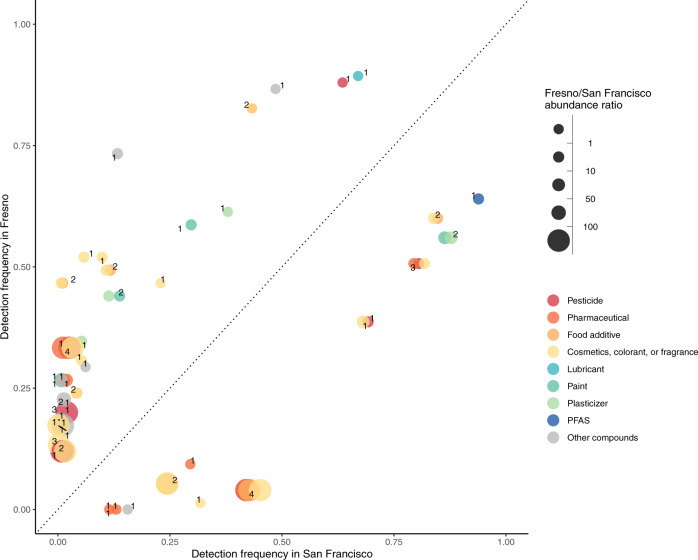
Differentially detected suspect chemicals, organized by source, in maternal serum between Fresno and San Francisco participants. Note: There were *N* = 75 participants in Fresno and *N* = 75 participants in San Francisco in this analysis. Any chemicals that were identified as potentially endogenous were removed from this plot. Individual chemicals may appear multiple times in multiple categories if they were present in databases for multiple categories. When this occurs the points are slightly jittered to show each of the categories. The number next to the chemicals indicate how many categories one chemical falls under, and therefore how many markers belong to each chemical. The size of each marker reflects the ratio of the average abundances across participants for each suspect chemical in Fresno compared to San Francisco. The median ratio of average abundances in Fresno compared to San Francisco was 9.7. All differentially detected chemicals had detection frequencies that were significantly different between cities at the 0.05 level.

**Fig. 4:**
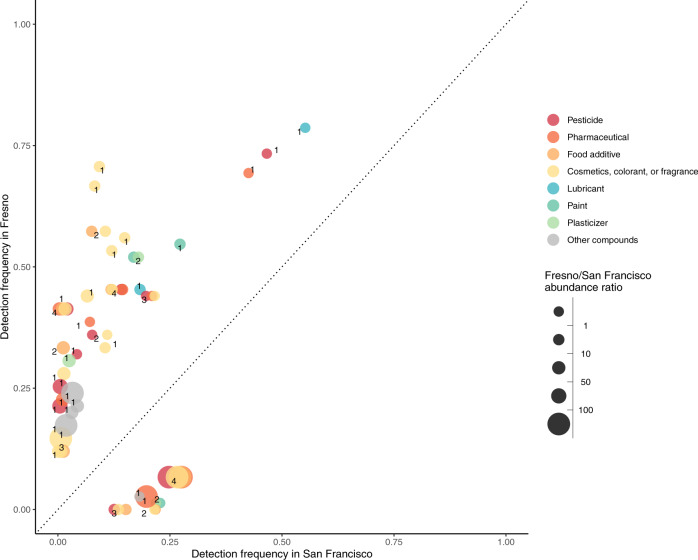
Differentially detected suspect chemicals, organized by source, in cord serum between Fresno and San Francisco participants. Note: There were *N* = 64 participants in Fresno and *N* = 64 participants in San Francisco in this analysis. Any chemicals that were identified as potentially endogenous were removed from this plot. Individual chemicals may appear multiple times in multiple categories if they were present in databases for multiple categories. When this occurs the points are slightly jittered to show each of the categories. The number next to the chemicals indicate how many categories one chemical falls under, and therefore how many markers belong to each chemical. The size of each marker reflects the ratio of the average abundances across participants for each suspect chemical in Fresno compared to San Francisco. The median ratio of average abundances in Fresno compared to San Francisco was 3.0. All differentially detected chemicals had detection frequencies that were significantly different between cities at the 0.05 level.

Of those suspect chemicals that were differentially detected between maternal samples in Fresno and San Francisco, there tended to be higher detection frequencies and abundances in Fresno (Fig. 3). The range of abundance ratios between Fresno and San Francisco was 0.003 to 1054.8.The category with the highest number of differentially detected chemicals are annotated as ingredients in cosmetics, colorants, or fragrances. However, one PFAS was detected in a higher proportion of maternal samples from San Francisco compared to Fresno. The higher rates of differentially detected chemicals in Fresno and abundances were similar for the neonatal samples (Fig. 4). The range of abundance ratios between Fresno and San Francisco cord samples was 0.0001 to 774.7. Results were very similar across all iterations of the matching procedure.

### Comparison of wristband exposure measurements and NTA

Among the 26 Fresno participants who wore silicone wristbands, 81 chemicals were detected out of the possible 1528 chemicals in the chemical screen (A complete list of the 81 chemicals detected in the wristbands, the detection frequencies and concentrations, their industrial or commercial uses, and the 2016 national aggregate production volumes is available in Supplementary Table 4). The chemicals could belong to more than one source category, but of the 14 chemicals detected in >90% of participants, 8 chemicals belonged to the chemicals in commerce group, 8 were in personal care products, 3 were pesticides, one was a flame retardant, and one was in the consumer products group (Supplementary Table 3). The chemicals detected in 50–90% of participants were diethyl phthalate, coumarin, di-n-nonyl phthalate, N, N-Diethyl-m-toluamide, B-citronellol, citral A, butylated hydroxyanisole, and amyl cinnamal.

**Fig. 5:**
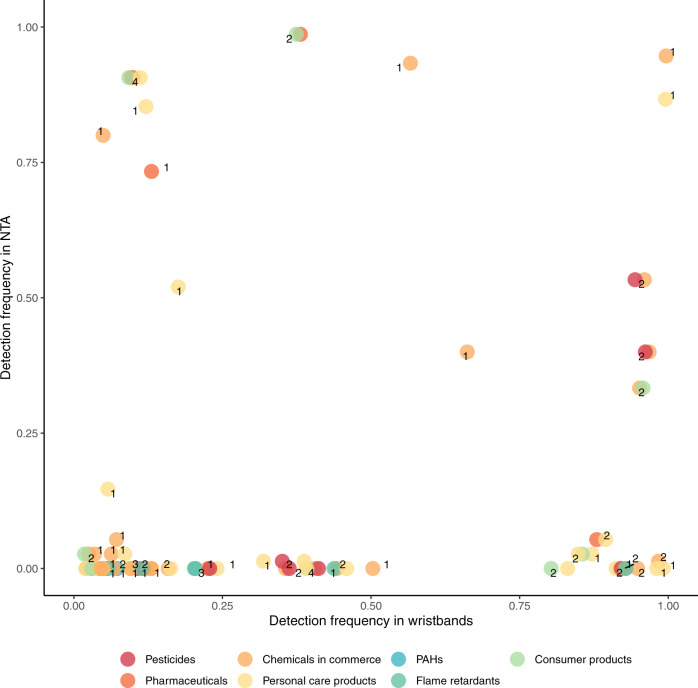
Detection frequencies of chemicals in silicone wristbands, organized by source, compared to detection in maternal serum among Fresno participants. Note: This analysis included data on *N* = 26 Fresno participants. Individual chemicals may appear in multiple categories. When this occurs the points are slightly jittered to show each of the categories. The number next to the chemicals indicate how many categories one chemical falls under, and therefore how many markers belong to each chemical. Chemicals were matched by formula and ranked detection frequency. Only chemicals that were present in databases for both the wristband and non-targeted analyses are included.

Of the total of 81 chemicals detected in the wristbands, there were 56 chemicals detected in the wristbands that could plausibly be detected in maternal serum using the non-targeted approach (because they were present in the database used in the non-targeted analysis). We detected more than half of these in maternal serum (31 of 56 possible). We found pharmaceuticals, chemicals in commerce, and personal care products were more likely to be detected across both exposure-assessment approaches. PAHs and flame retardants were detected in the wristbands but not in maternal serum (Fig. 5). The relationship was weak between the abundances in maternal serum and the ng/g silicone per week in the wristbands (rho = 0.08, *p* value = 0.16) (Supplementary Fig. 6).

### Comparison of geographic measures of exposure from CalEnviroScreen with NTA

**Fig. 6:**
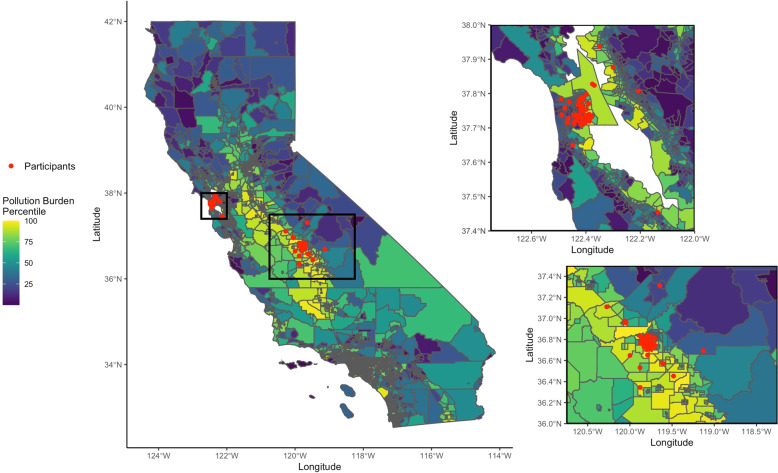
Maps of Fresno and San Francisco participants linked to CalEnviroScreen Pollution Burden percentiles. Note: The participant locations have been jittered slightly to protect participant confidentiality.

Although most participants delivering in Fresno lived close to the city center, some participants lived in more rural areas of Fresno County or in the surrounding cities of the Bay Area (Fig. 6). Fresno participants lived in tracts with higher CalEnviroScreen pollution burden percentiles compared to the San Francisco participants (Supplementary Table 1), but these scores were negatively correlated with the overall number of chemicals detected in maternal serum among the Fresno participants (rho = −0.21, *p* value = 0.08) and not correlated in San Francisco participants (rho = −0.03, *p* value = 0.80) (Supplementary Fig 7). Living in an area of Fresno County with higher CalEnviroScreen Pesticide percentiles was correlated with the number of differentially detected suspect chemicals annotated as pesticides in maternal serum (rho = 0.33, *p* value < 0.005) (Supplementary Fig 7). Only a few San Francisco participants live in areas where there were pesticides used for agricultural production, and the correlation between the Pesticide percentiles and the number of differentially detected suspect chemicals annotated as pesticides was smaller and not significant (rho = 0.10, *p* value = 0.39). The abundances of three of the eleven suspect pesticides differentially detected in maternal serum were significantly correlated with the Pesticide percentiles (Supplementary Fig. 8).

## Discussion

This comprehensive comparison of prenatal exposures from non-targeted analysis on maternal and neonatal sera, targeted analysis of silicone wristbands, and area-level measures of exposure better characterizes the prenatal exposome among pregnant women living near San Francisco and Fresno, California. While we observed a similar number of chemicals detected in maternal and cord sera samples from both places, a higher number of chemicals annotated as pesticides, food additives, cosmetics, colorants, and fragrances were observed among Fresno pregnant women compared to San Francisco. More than half of the chemicals detected in the wristbands were also found using the NTA approach, although the abundances and concentrations in matched samples were not strongly correlated. The lack of correlation is not surprising given the small sample size, the limited overlap in chemical space between GC and LC, and because we had to link the datasets using only the chemical formula. We observed a correlation between the CalEnviroScreen geographic measure of pesticide exposure and the number of pesticides differentially detected among the Fresno participants, indicating that the NTA analysis can capture sources of exposure and that high pesticide use in production agriculture can increase the body burden of pesticides among pregnant women living nearby. However, the pollution burden exposure metric was not correlated with the number of suspect chemicals detected in maternal serum in either San Francisco or Fresno.

We found widespread exposure to multiple chemicals, some of which are not routinely measured in pregnant women and children. For instance, 100% of the Fresno participants who wore wristbands were exposed to several chemicals used in fragrances, including ethylene brassylate, benzyl salicylate, tonalide, beta-ionone, and lilial (Supplementary Table 3). While there is evidence of developmental toxicity for one of these chemicals, benzyl salicylate [34], the others have no or limited evidence insufficient to characterize their to affect human development [35–37]. Two phthalates used as plasticizers, diisobutyl phthalate (DIBP) and di-n-butyl phthalate (DBP), were also detected in 100% of the women wearing wristbands, both of which are known reproductive and developmental toxicants [38–41]. DBP is banned for use in children’s toys based on evidence of toxicity, and a ban of DIBP has been proposed [42]. Phthalates are comparatively very well-studied compared to the other chemicals in fragrances that we identified. This may be due to a lack of policies and regulation in the United States for disclosure of ingredients in any fragrance, sufficient testing to identify potential health effects, and the confidential nature of fragrance formulations for industry [43].

There were several limitations to our approach. The serum samples from San Francisco and Fresno were analyzed in separate batches; therefore, it is difficult to assess whether observed differences are due to actual exposure differences or batch effects. However, we used a conservative approach for removing batch effects; therefore, there may have been actual differences between the Fresno and San Francisco groups that we were unable to detect. To address the differences in maternal characteristics across Fresno and San Francisco, we used nearest neighbor matching based on propensity scores for living in Fresno. While the distribution of characteristics was much more similar after matching, there were still some differences across groups as San Francisco participants were older on average; therefore, there may be residual confounding by age in our study. Additionally, while non-targeted approaches provide a comprehensive way to screen for a very large spectrum of chemical exposures, often the vast majority of the detected chemical features remain as detected masses or just assigned formulas without specific structural information or actual concentrations. It is, thus, important to acknowledge that when working with chemical features that were not confirmed with analytical standards or matched to MS/MS spectral libraries there is always the possibility of a wrongly assigned structure.

It is possible the silicone wristbands have higher retention of certain types of chemical exposures compared to concentrations in maternal serum. For instance, some chemicals may be present in the air or primarily excreted through sweat, and therefore recorded in the wristbands, but not present in high concentrations in blood due to the mechanisms of metabolism or excretion that affect the biological half-life of those chemicals. For instance, we only measured chemicals in serum, and certain chemicals like some flame retardants and PAHs are better detected in urine. Additionally, non-targeted analyses have lower sensitivity compared to targeted approaches. Therefore, the difference in sensitivities of the two exposure-assessment approaches may explain some of the discrepancies we observed in detection frequencies of certain chemicals between the wristbands and non-targeted analysis of maternal sera. The non-targeted analysis utilized a larger library than the wristband approach, so there may have been chemicals present in the wristbands that we were not able to measure using the narrower targeted chemical screen. Additionally, some chemicals in the wristband database were not included in the non-targeted database, such as PCBs and PBDEs, because these chemicals were not amenable to liquid chromatography. For example, 207 of the 1528 potential compounds detectable in wristbands were PCBs, and 59 were PBDEs. Due to the differences in methods between the identification of chemicals in the wristbands and the biospecimens, targeted analysis or GC-QTOF-MS instead of LC-QTOF-MS would be necessary to more accurately compare detection frequencies of specific chemicals between the two approaches.

This study illustrates how residential geography can affect the differential chemical burdens that pregnant women and their children are exposed to in utero. These exposures can affect health during pregnancy and influence long-term health of both mother and child. Our analyses illustrate a more comprehensive approach to exposure assessment, including non-targeted analyses and passive exposure-assessment approaches like silicone wristbands can more fully capture the human exposome. The current U.S. regulatory environment which allows chemicals to be on the marketplace without testing contributes to universal exposures with unknown health effects, which is especially worrisome for exposures among pregnant women and children. While future research should focus on identifying health effects of these chemicals, approaches that require better exposure source information and pre-exposure identification of health harms is needed.

## Supplementary information

Supplementary information

## Data Availability

The Fresno Biomonitoring Study and Chemicals in Our Bodies-2 datasets analysed in the current study are not publicly available due to participant privacy. The CalEnviroScreen data is publicly available at https://oehha.ca.gov/calenviroscreen/report/calenviroscreen-30.
