# Supplementary material for: Investigating geographic differences in environmental chemical exposures in maternal and cord sera using non-targeted screening and silicone wristbands in California
Source: J Expo Sci Environ Epidemiol. 2022 Apr 21;33(4):548–57. doi: 10.1038/s41370-022-00426-9 (PMC9585116; doi:10.1038/s41370-022-00426-9)
Supplement: Supplementary file 1 — Supplementary information [file 41370_2022_426_MOESM1_ESM.docx]

Supplemental material for **Investigating geographic differences in environmental chemical exposures in maternal and cord sera using non-targeted screening and silicone wristbands in California**

Table of contents

Additional QA/QC information …………………………………..………………………………..…………………… 2-3

Supplemental Figure 1 …………………………………..…………………………………..…………………………….. 4

Supplemental Figure 2 …………………………………..…………………………………..…………………………….. 5

Supplemental Figure 3 …………………………………..…………………………………..…………………………….. 6

Supplemental Figure 4 …………………………………..…………………………………..…………………………….. 7

Supplemental Figure 5 …………………………………..…………………………………..…………………………….. 8

Supplemental Figure 6 …………………………………..…………………………………..…………………………….. 9

Supplemental Figure 7 …………………………………..…………………………………..…………………………….. 10

Supplemental Figure 8 …………………………………..…………………………………..…………………………….. 11

Supplemental Table 1 .…………………………………..…………………………………..…………………………….. 12

Supplemental Table 2 .…………………………………..…………………………………..…………………………….. 13

Supplemental Table 3 .…………………………………..…………………………………..…………………………….. 14-15

Supplemental Table 4 .…………………………………..…………………………………..…………………………….. 17-38

**Additional QA/QC information.**

Isotope labeled standards were used as surrogate, among which M2PFOA was monitored in the negative mode, and D15-Triphenyl Phosphate (D15-TPP) and DL-Cotinine were used in the positive mode. The mass accuracy and retention time consistency were monitored. In addition, QC material (7 OPFRs and 10 PFASs spiked blank serum) was injected with every batch of samples and monitored.

| Surrogate | Formula | Pos *m/z* | Neg *m/z* |
| --- | --- | --- | --- |
| M2PFOA | [13C]2C6HF15O2 |  | 414.9725 |
| Triphenyl Phosphate (D15-TPP) | C18D15O4P | 342.1728 |  |
| DL-Cotinine (methyl D3) | C10H9D3N2O | 180.1216 |  |

The water blank was used as procedural blank, and only features that were 2 times or higher in samples were retained. Spiked Matrix was used as QC material. 7 OPFRs and 10 PFASs were spiked into blank serum and analyzed alongside the samples. The spiked standards were monitored between the batches for consistency. Please see below table for detailed information.

| Name | Formula (MS Ready) | Mass | Experimental Retention Time | CAS | ionization |
| --- | --- | --- | --- | --- | --- |
| Perfluoro-n-butanoic acid (PFBA) | C4HF7O2 | 213.98648 | 4.1 | 375-22-4 | neg |
| Perfluoro-n-pentanoic acid (PFPA) | C5HF9O2 | 263.98328 | 5.9 | 2706-90-3 | neg |
| Perfluoro-n-hexanoic acid (PFHxA) | C6HF11O2 | 313.98009 | 7.1 | 307-24-4 | neg |
| Perfluoro-n-heptanoic acid (PFHpA) | C7HF13O2 | 363.9769 | 8.4 | 375-85-9 | neg |
| Perfluoro-n-octanoic acid (PFOA) | C8HF15O2 | 413.9737 | 9.6 | 335-67-1 | neg |
| Perfluoro-n-nonanoic acid (PFNA) | C9HF17O2 | 463.97051 | 10.8 | 375-95-1 | neg |
| Perfluoro-n-decanoic acid (PFDA) | C10HF19O2 | 513.96732 | 11.8 | 335-76-2 | neg |
| Potassium perfluoro-1-butanesulfonate (L-PFBS) | C4HF9O3S | 299.95027 | 6.1 | 29420-49-3 | neg |
| Sodium perfluoro-1-hexanesulfonate (L-PFHxS) | C6HF13O3S | 399.94388 | 8.4 | 82382-12-5 | neg |
| Sodium perfluoro-1-octanesulfonate (L-PFOS) | C8HF17O3S | 499.93749 | 10.7 | 4021-47-0 | neg |
| Tris(2-chloroethyl) phosphate | C6H12Cl3O4P | 283.95388 | 6.749 | 115-96-8 | pos |
| Tris(2-chloroisopropyl) phosphate | C9H18Cl3O4P | 326.00083 | 9.598 | 13674-84-5 | pos |
| Tris(1,3-dichloro-2-propyl) phosphate | C9H15Cl6O4P | 427.88391 | 11.776 | 13674-87-8 | pos |
| Triethyl phosphate | C6H15O4P | 182.0708 | 5.912 | 4021-47-2 | pos |
| Tripropyl phosphate | C9H21O4P | 224.11775 | 9.46 | 513-08-06 | pos |
| Tributyl phosphate | C12H27O4P | 266.1647 | 12.949 | 4021-47-1 | pos |
| Triphenyl phosphate | C18H15O4P | 326.0708 | 11.943 | 4021-47-3 | pos |

**Supplemental Figure 1.** Distribution of propensity scores for living in Fresno among all participants before restriction.

**Supplemental Figure 2.** Distribution of propensity scores for living in Fresno among maternal samples after restriction.

**Supplemental Figure 3.** Distribution of propensity scores for living in Fresno among neonatal samples after restriction.

**Supplemental Figure 4.** Comparison of log average chemical abundances in maternal serum in Fresno and San Francisco.

**
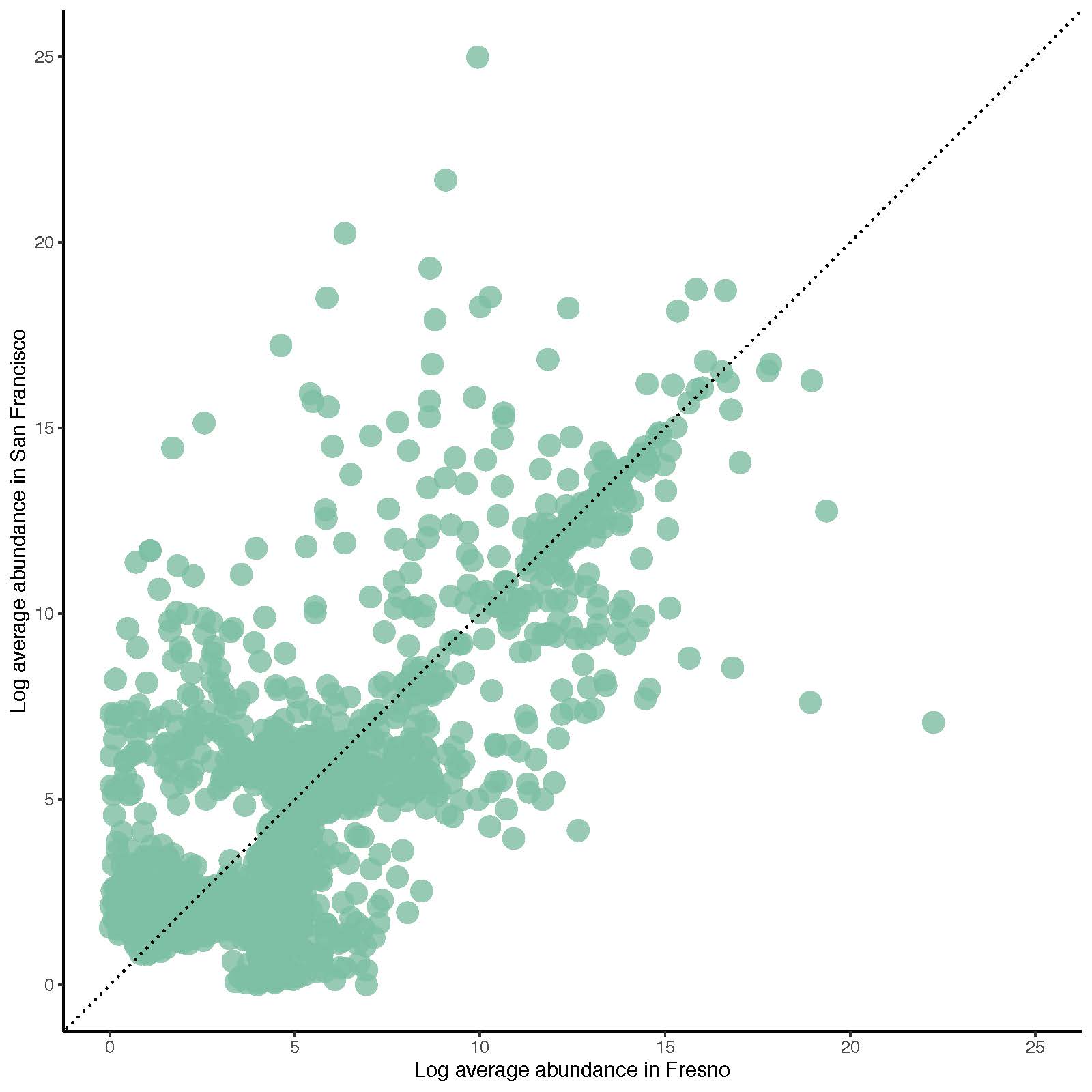
**

Note: The log average abundances between cities were correlated (Pearson correlation coefficient = 0.71, p-value <0.001). San Francisco participants had higher log average abundances (average among San Francisco participants was 4.43 versus 3.95 among Fresno participants, t-test p-value <0.001).

**Supplemental Figure 5.** Comparison of log average chemical abundances in cord serum in Fresno and San Francisco.

**
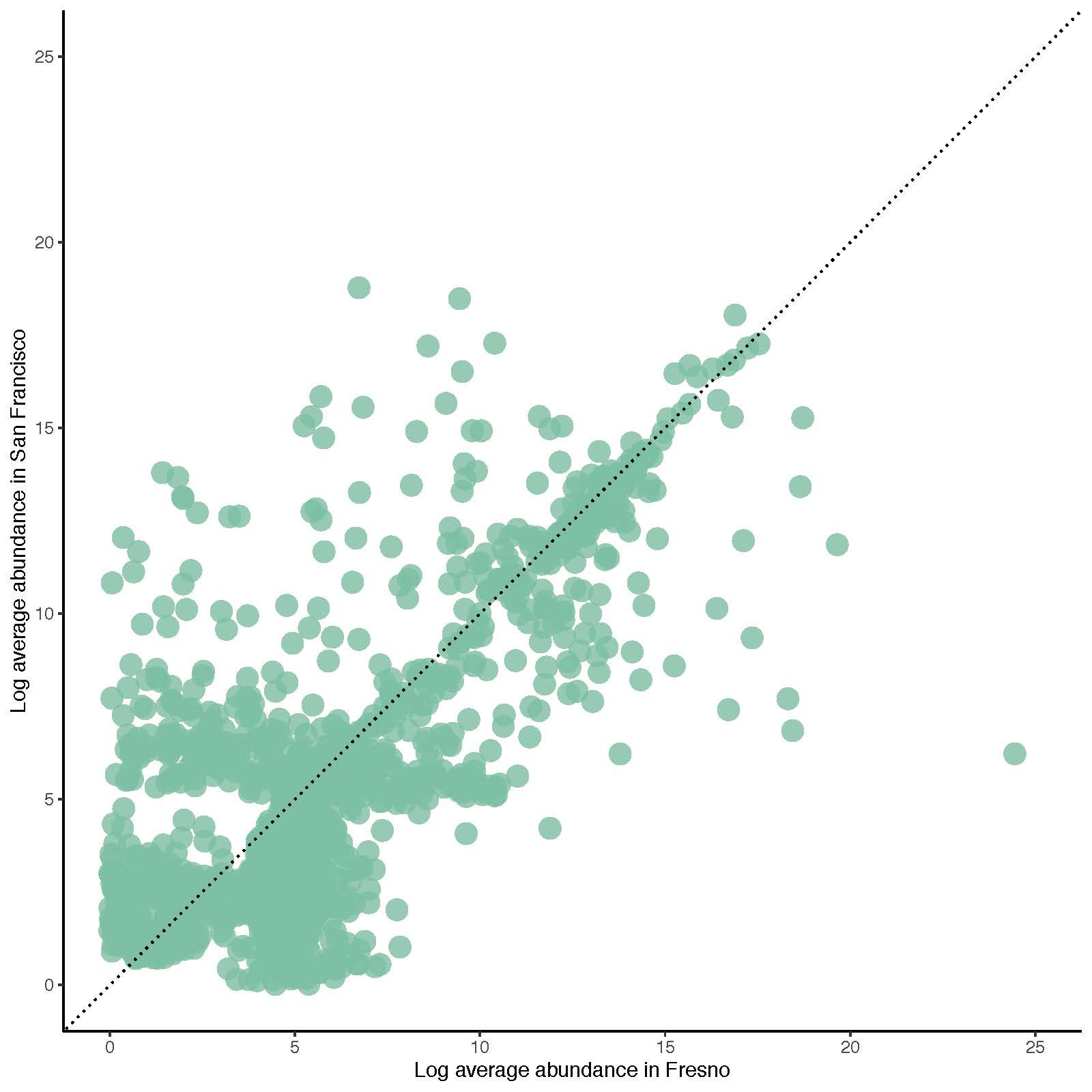
**

Note: The log average abundances between cities were correlated (Pearson correlation coefficient = 0.71, p-value <0.001). San Francisco participants had higher log average abundances (average among San Francisco participants was 4.47 versus 3.98 among Fresno participants, t-test p-value <0.001).

**Supplemental Figure 6.** Correlation between chemical abundances in maternal serum and ng/g silicone per week in wristbands worn by the same participants for chemicals detected in wristbands whose formulas were also detected using non-targeted analyses.

Note: Only chemicals with unique formulas present in both the wristbands and non-targeted analysis were included, which resulted in 26 chemicals for each of the 26 participants who wore wristbands and had NTA results. The Spearman’s correlation coefficient was: rho=0.08, p-value=0.16.

**
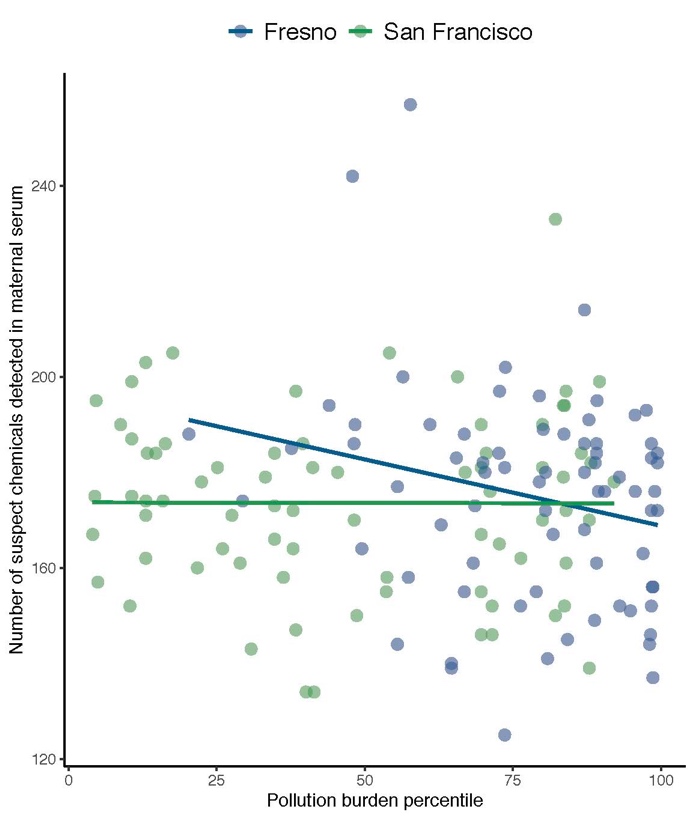
Supplemental Figure 7**. Correlation between geographic measure of pollution using CalEnviroScreen Pollution Burden and Pesticide Percentile and number of suspect chemicals or suspect chemicals differentially detected by city in maternal serum and annotated as pesticides**.**

**
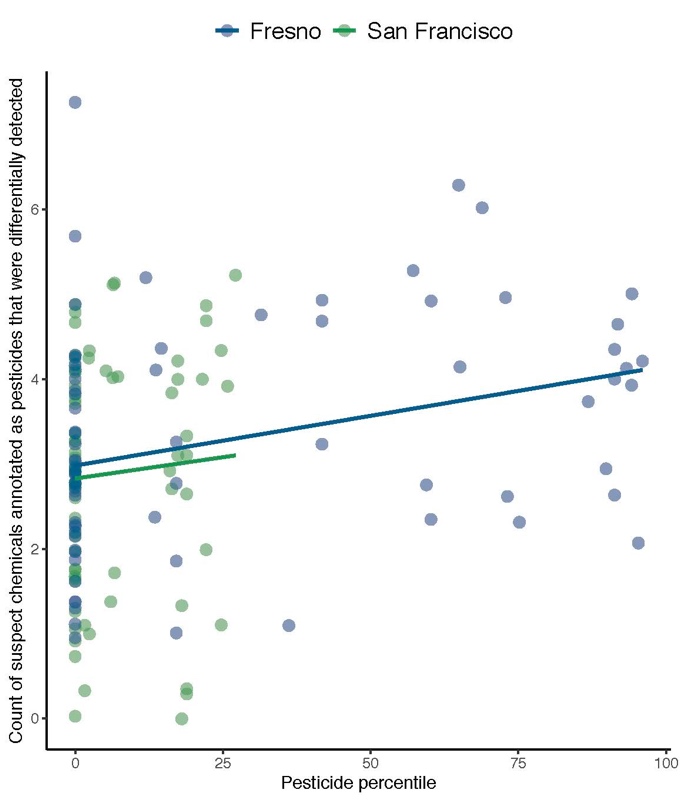
**

Note: The lines are the linear regression fit for each city. The Spearman’s correlation between the Pollution burden percentile and the number of suspect chemicals detected is rho= -0.21, p-value =0.08 for the Fresno participants and rho = -0.03, p-value = 0.80 for the San Francisco participants. The Spearman’s correlation between the Pesticide percentile and the number of suspect chemicals annotated as pesticides that were differentially detected was rho=0.33, p-value <0.005 for the Fresno participants and rho=0.10, p-value=0.39 for the San Francisco participants.

**Supplemental Figure 8**. Comparison between CalEnviroScreen Pesticide Percentile and abundances of suspect pesticides differentially detected by city in maternal serum**.**


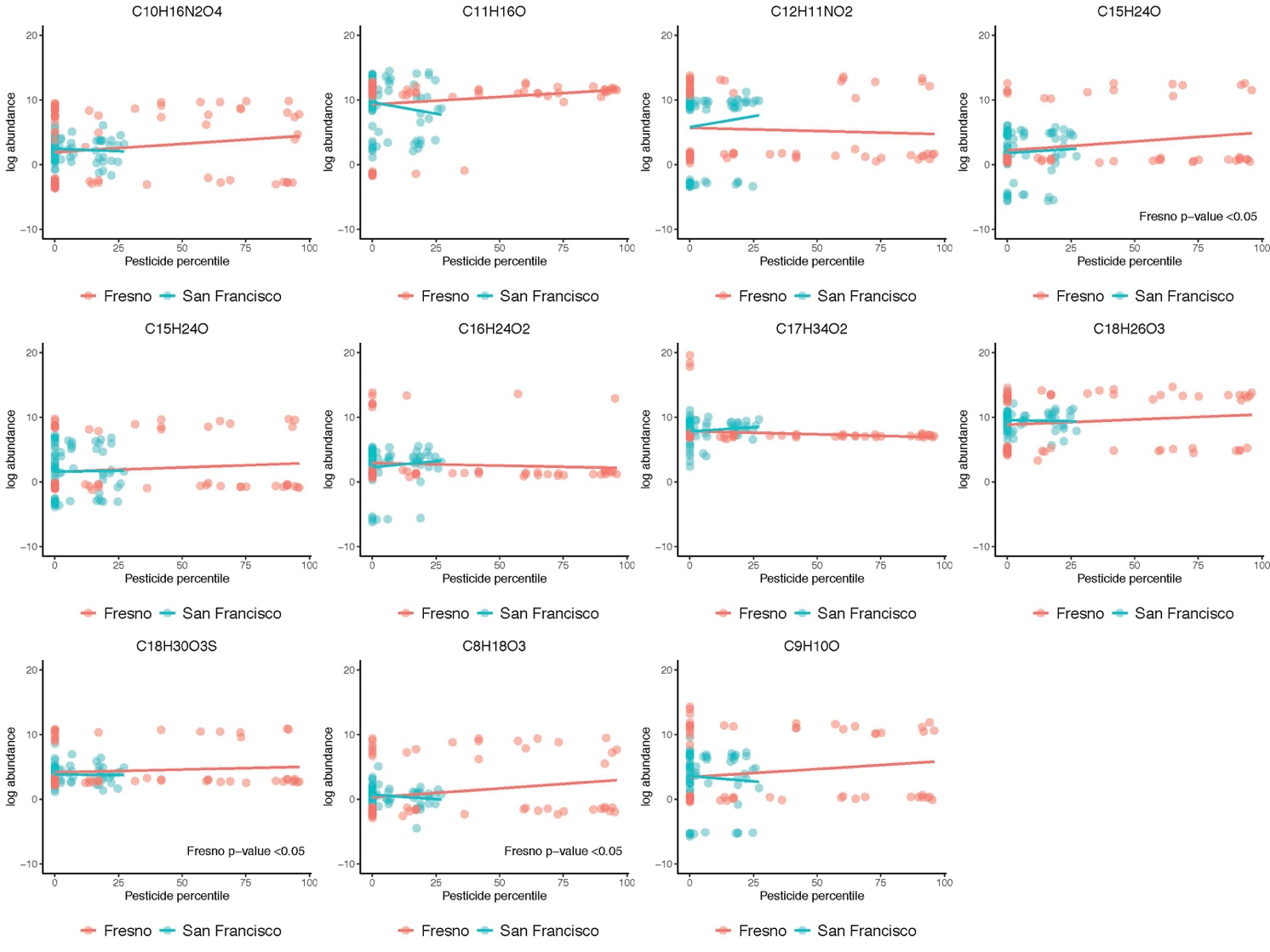


Note: The lines are the linear regression fit for each city. The formulas for each suspect pesticide that was differentially detected between cities is shown at the top of each plot. If the correlation between the pesticide percentiles and the abundances in maternal serum by city of residence was statistically significant, this is noted in the bottom of each plot.

**Supplemental Table 1.** Descriptive statistics of study participants with maternal and cord serum in Fresno and San Francisco before and after matching.

| Participant demographics | **Maternal samples** | | | **Neonatal samples** | | |
| --- | --- | --- | --- | --- | --- | --- |
|  | **Fresno** | **San Francisco** | | **Fresno** | **San Francisco** | |
| N (%) | (N=75) | Before matching (N=294) | After matching (N=75) | (N=64) | Before matching (N=294) | After matching (N=64) |
| **Age** |  |  |  |  |  |  |
| 15-25 | 31 (41) | 26 (9) | 25 (33) | 26 (41) | 26 (9) | 25 (39) |
| 25-30 | 29 (39) | 41 (14) | 20 (27) | 25 (39) | 41 (14) | 19 (30) |
| 30-35 | 12 (16) | 134 (46) | 19 (25) | 11 (17) | 134 (46) | 14 (22) |
| 35-47 | 3 (4) | 93 (32) | 11 (15) | 2 (3) | 93 (32) | 6 (9) |
| **Educational attainment** |  |  |  |  |  |  |
| Less than high school | 27 (36) | 26 (9) | 18 (24) | 23 (36) | 26 (9) | 16 (25) |
| High school diploma or GED | 25 (33) | 44 (15) | 30 (40) | 22 (34) | 44 (15) | 27 (42) |
| Some college course work for credit or AA degree | 22 (29) | 29 (10) | 25 (33) | 18 (28) | 29 (10) | 20 (31) |
| Bachelor's degree (4 years) | 1 (1) | 195 (66) | 2 (3) | 1 (2) | 195 (66) | 1 (2) |
| **Race/ethnicity** |  |  |  |  |  |  |
| White | 6 (8) | 127 (43) | 10 (13) | 5 (8) | 127 (43) | 8 (13) |
| Latina | 55 (73) | 91 (31) | 51 (68) | 47 (73) | 91 (31) | 47 (73) |
| Black | 6 (8) | 14 (5) | 8 (11) | 5 (8) | 14 (5) | 6 (9) |
| Asian/PI | 4 (5) | 47 (16) | 4 (5) | 4 (6) | 47 (16) | 2 (3) |
| Other or multiple | 4 (5) | 15 (5) | 2 (3) | 3 (5) | 15 (5) | 1 (2) |
| **Marital status*** |  |  |  |  |  |  |
| Married | 56 (75) | 214 (73) | 34 (45) | 47 (73) | 214 (73) | 27 (42) |
| Divorced or separated | 2 (3) | 9 (3) | 3 (4) | 1 (2) | 9 (3) | 1 (2) |
| Never married | 17 (23) | 71 (24) | 38 (51) | 16 (25) | 71 (24) | 36 (56) |
| **CalEnviroScreen** [median (IQR)] |  |  |  |  |  |  |
| Pollution burden percentile | 80.8  (66.8-93.0) | 33.6  (14.5-64.4) | 41.5  (22.1-74.5) | 80.7  (68.4 – 95.6) | 33.6  (14.5-64.4) | 40.7  (21.6 – 71.5) |
| Pesticide burden percentile | 0  (0-60.2) | 0  (0-0) | 0  (0-16.4) | 12.0  (0 – 63.7) | 0  (0-0) | 0  (0 – 16.4) |

* In the Fresno questionnaire, women were asked if they were married or partnered. In the San Francisco questionnaire, women were asked if they were married. Percentages may not sum to exactly 100% due to rounding.

**Supplemental Table 2**. Comparison of Fresno participant demographics in total sample used in non-targeted analysis versus wristbands.

| Participant demographics | **Non-targeted analyses** | **Wristband analyses** |
| --- | --- | --- |
| N (%) | (N=75) | (N=26) |
| **Age** |  |  |
| 15-25 | 31 (41) | 11 (42) |
| 25-30 | 29 (39) | 9 (35) |
| 30-35 | 12 (16) | 4 (15) |
| 35-47 | 3 (4) | 2 (8) |
| **Educational attainment** |  |  |
| Less than high school | 27 (36) | 8 (31) |
| High school diploma or GED | 25 (33) | 10 (39) |
| Some college course work for credit or AA degree | 22 (29) | 8 (31) |
| Bachelor's degree (4 years) | 1 (1) | 0 (0) |
| **Race/ethnicity** |  |  |
| White | 6 (8) | 4 (15) |
| Latina | 55 (73) | 18 (69) |
| Black | 6 (8) | 2 (8) |
| Asian/PI | 4 (5) | 1 (4) |
| Other or multiple | 4 (5) | 1 (4) |
| **Marital status*** |  |  |
| Married | 56 (75) | 17 (65) |
| Divorced or separated | 2 (3) | 1 (4) |
| Never married | 17 (23) | 8 (31) |

* In the Fresno questionnaire, women were asked if they were married or partnered. In the San Francisco questionnaire, women were asked if they were married. Percentages may not sum to exactly 100% due to rounding.

**Supplemental Table 3.** Chemicals detected in >90% of wristbands worn by study participants in Fresno (N=26).

| **Chemical**  **(CASRN)** | **Category^1^** | **Specific Uses or Sources of Exposure^2^** | **2016 National aggregate production volume^3^** | **Percent detected** | **Median ng/g silicone per week among those detected** | **Average ng/g silicone per week among those detected** | **Minimum ng/g silicone per week among those detected** | **Maximum ng/g silicone per week among those detected** |
| --- | --- | --- | --- | --- | --- | --- | --- | --- |
| Di-n-butyl phthalate^4^  (84-74-2) | personal care, chemicals in commerce, pesticides | • plasticizer | 1,000,000 - 10,000,000 lb | 100% | 1560 | 1875 | 235 | 7200 |
| Ethylene brassylate  (105-95-3) | personal care | • fragrance  • cleaning products  • food additive  • personal care products  • pesticides | 500,000 - 1,000,000 lb | 100% | 1500 | 3177 | 22 | 23200 |
| Diisobutyl phthalate^4^  (84-69-5) | chemicals in commerce | • plasticizer • personal care products • concrete additive | <1,000,000 lbs | 100% | 1320 | 2097 | 440 | 12600 |
| Benzyl salicylate  (118-58-1) | personal care | • fragrance  • food additive | 1,000,000 - 10,000,000 lb | 100% | 1070 | 2224 | 17 | 18000 |
| Lilial  (80-54-6) | personal care | • cleaning products  • fragrance  • personal care products  • pesticides | 500,000 - 1,000,000 lb | 100% | 155 | 193 | 35 | 586 |
| Tonalide  (1506-02-1) | personal care | •fragrance | <1,000,000 lbs | 100% | 73 | 102 | 7 | 540 |
| B-Ionone  (79-77-6) | personal care | • fragrance  • food additive  • cigarettes  • cleaning products  • personal care products | **-** | 100% | 37 | 64 | 3 | 548 |
| Galaxolide  (1222-05-5) | personal care, chemicals in commerce | • fragrance | 1,000,000 - 10,000,000 lb | 96% | 1965 | 2533 | 78 | 11700 |
| Butyl benzyl phthalate  (85-68-7) | chemicals in commerce | • commercial products | 10,000,000 - 50,000,000 lb | 96% | 757 | 973 | 73 | 4950 |
| Bis(2-ethylhexyl)phthalate  (117-81-7) | chemicals in commerce, pesticides | •plasticizer  •medical devices | 10,000,000 - 50,000,000 lbs | 96% | 215 | 1873 | 76 | 26400 |
| Butylated hydroxytoluene^4^  (128-37-0) | chemicals in commerce, consumer products | • food additive • personal care products  • commercial products | 10,000,000 - 50,000,000 lb | 96% | 68 | 115 | 8 | 955 |
| Benzyl benzoate  (120-51-4) | pesticides | • plastics • food additive • pesticides | 1,000,000 - 10,000,000 lb | 93% | 809 | 1049 | 210 | 3990 |
| Benzophenone  (119-61-9) | chemicals in commerce, personal care | • pharmaceuticals  • pesticides • fragrance  • cigarettes  • cleaning products  • food additive  • personal care products  • commercial products | 1,000,000 - 10,000,000 lb | 93% | 54 | 98 | 8 | 452 |
| meso-Tetra(4-carboxyphenyl)porphine  (26248-87-3) | chemicals in commerce, flame retardant | • flame retardant • fragrance • pesticides | **-** | 93% | 96 | 155 | 12 | 735 |

^1^Categories were determined based on chemical structure and source data from the hazardous substances database from the national library of medicine (<https://pubchem.ncbi.nlm.nih.gov/>).

^2^Specific Uses and Sources of Exposure were determined based on the Use and Manufacturing sections from the national library of medicine (<https://pubchem.ncbi.nlm.nih.gov/>).

^3^Production volumes for 2016 were collected from the EPA Chemical Reporting Database (<https://www.epa.gov/chemical-data-reporting/access-cdr-data#2016>). Any chemicals with no production volume listed were not present in the database.

^4^Blank wristbands run within this analysis showed low level detections of butylated hydroxytoluene, diethyl phthalate, di-n-butyl phthalate and diisobutyl phthalate; however, no detection in any blank exceeded 25% of the lowest reported value for these analytes.

**Supplemental Table 4.** All chemicals detected in silicone wristbands, organized by detection frequency.

| **Chemical** | **Category** | **Fragrance-related** | **Food additive or flavoring** | **Pesticide-related** | **Specific Uses** | **CASRN** | **% detected among Fresno women** | **Average ng/g silicone per week among those detected** | **Minimum ng/g silicone per week among those detected** | **Maximum ng/g silicone per week among those detected** | **Median ng/g silicone per week among those detected** | **2016 National aggregate production volume** |
| --- | --- | --- | --- | --- | --- | --- | --- | --- | --- | --- | --- | --- |
| **Ethylene brassylate** | personal care | Yes | Yes | Yes | • fragrance  • used in cleaning products  • food additive  • found in personal care products  • found in pesticides  Source: https://pubchem.ncbi.nlm.nih.gov/compound/61014#section=Uses | 105-95-3 | 100% | 3176.9 | 22.0 | 23200.0 | 1500.0 | 500,000 - 1,000,000 lb |
| **Benzyl salicylate** | personal care | Yes | Yes | No | •fragrance in soaps, cosmetics, sunscreen lotions, deodorants, household air fresheners •food additive Source: https://pubchem.ncbi.nlm.nih.gov/compound/8363#section=Use-and-Manufacturing | 118-58-1 | 100% | 2223.7 | 17.1 | 18000.0 | 1070.0 | 1,000,000 - 10,000,000 lb |
| **Tonalide** | personal care | Yes | No | No | •fragrance in soaps and cosmetics •previously used in cleaners & detergents Source: https://pubchem.ncbi.nlm.nih.gov/compound/89440#section=Overview | 1506-02-1 | 100% | 102.0 | 7.3 | 540.0 | 73.2 | <1,000,000 lbs |
| **B-Ionone** | personal care | Yes | Yes | No | • used as a fragrance  • used as a food flavoring  • used in cigarettes  • present in household cleaning products  • found in personal care products  Source: https://pubchem.ncbi.nlm.nih.gov/compound/638014#section=Uses | 79-77-6 | 100% | 64.1 | 3.1 | 548.0 | 37.2 | **-** |
| **Lilial** | personal care | Yes | No | Yes | • used in air fresheners  • used in cleaning products  • used in fragrance  • used in personal care products  • inert ingredient in pesticides  Source: https://pubchem.ncbi.nlm.nih.gov/compound/228987#section=Uses | 80-54-6 | 100% | 193.1 | 35.2 | 586.0 | 155.0 | 500,000 - 1,000,000 lb |
| **Diisobutyl phthalate** | chemicals in commerce | No | No | No | •plasticizer •used in nail polish, cosmetics, lubricants, floor carpets, tapestry, clothing treatments, rubber dentistry settings, fuel stabilizer, in leather varnishes and lacquers •concrete additive Source***:*** https://pubchem.ncbi.nlm.nih.gov/compound/6782#section=Uses | 84-69-5 | 100% | 2097.1 | 440.0 | 12600.0 | 1320.0 | <1,000,000 lbs |
| **Di-n-butyl phthalate** | personal care, chemicals in commerce, pesticides | No | No | Yes | •plasticizer • used in PVC piping, carpet backing, paints, varnishes, lacquers, medical supplies, safety glass for automobiles, food packaging, cosmetics, textiles, and paper coatings.  Source**:** https://pubchem.ncbi.nlm.nih.gov/compound/3026#section=Uses | 84-74-2 | 100% | 1874.8 | 235.0 | 7200.0 | 1560.0 | 1,000,000 - 10,000,000 lb |
| **Bis(2-ethylhexyl)phthalate** | chemicals in commerce, pesticides | No | No | Yes | •plasticizer found in tablecloths, shower curtains, furniture, automobile upholstery, imitation leather, garden hoses, floor tiles, swimming-pool liners, sheathing for wire and cable, rainwear, shoes, toys, dolls, food packaging materials, tubing used in commercial milking equipment, and weather stripping •used for medical devices including blood and intravenous solution bags, catheters, tubing for dialysis and intravenous solutions, oxygen masks, and urine and colostomy bags. Source: https://pubchem.ncbi.nlm.nih.gov/compound/8343#section=Use-and-Manufacturing | 117-81-7 | 96% | 1873.4 | 76.2 | 26400.0 | 215.0 | 10,000,000 - 50,000,000 lbs |
| **Galaxolide** | personal care, chemicals in commerce | Yes | No | No | •fragrance in perfumes, soaps, cosmetics, air fresheners, cleaning products Source: https://pubchem.ncbi.nlm.nih.gov/compound/91497#section=Overview | 1222-05-5 | 96% | 2533.0 | 78.2 | 11700.0 | 1965.0 | 1,000,000 - 10,000,000 lb |
| **Butylated hydroxytoluene** | chemicals in commerce, consumer products | No | Yes | No | •preservative in foods, cosmetics, personal care products, paints, inks, animal feeds, and many commercial products ***Source*:** https://pubchem.ncbi.nlm.nih.gov/compound/31404#section=Uses | 128-37-0 | 96% | 114.9 | 7.7 | 955.0 | 67.6 | 10,000,000 - 50,000,000 lb |
| **Butyl benzyl phthalate** | chemicals in commerce | No | No | No | •used in the production of vinyl tiles •used in food conveyor belts, carpet tile, artificial leather, tarps, automotive trim, weather stripping and traffic cones •found in some vinyl gloves, adhesive, and caulking products Source**:** https://pubchem.ncbi.nlm.nih.gov/compound/2347#section=Use-and-Manufacturing | 85-68-7 | 96% | 973.0 | 73.3 | 4950.0 | 757.0 | 10,000,000 - 50,000,000 lb |
| **Benzophenone** | chemicals in commerce, personal care | Yes | Yes | Yes | • used in manufacture of pharmaceuticals  • used in manufacture of pesticides • fragrance  • found in cigarettes  • found in home cleaning products  • colorant  • found in electronics  • related to food production and service  • food additive  • found in paint  • found in personal care products  • inert ingredient in pesticide  Source: https://pubchem.ncbi.nlm.nih.gov/compound/3102#section=Uses | 119-61-9 | 93% | 97.7 | 8.0 | 452.0 | 54.1 | 1,000,000 - 10,000,000 lb |
| **Benzyl benzoate** | pesticides | Yes | Yes | Yes | •plastics •food flavoring •perfume production •skin treatment in humans and dogs for mites •dust mite control Source: https://pubchem.ncbi.nlm.nih.gov/compound/Benzyl-benzoate#section=Uses | 120-51-4 | 93% | 1049.4 | 210.0 | 3990.0 | 809.0 | 1,000,000 - 10,000,000 lb |
| **TCPP** | chemicals in commerce, flame retardant | Yes | No | Yes | • flame retardant • fragrance • used in pesticides  Source: https://pubchem.ncbi.nlm.nih.gov/compound/14034#section=Use-and-Manufacturing | 26248-87-3 | 93% | 154.9 | 12.2 | 735.0 | 95.5 | **-** |
| **Amyl cinnamal** | personal care | Yes | Yes | Yes | • used in air fresheners • found in cigarettes  • found in household cleaning products  • found in food productive and service  • food additive  • fragrance • used in manufacturing  • used in personal care products  • pesticide  Source: https://pubchem.ncbi.nlm.nih.gov/compound/31209#section=Uses | 122-40-7 | 89% | 146.2 | 19.5 | 518.0 | 117.0 | 1,000,000 - 10,000,000 lb |
| **Butylated hydroxyanisole** | pharmacological, personal care | Yes | Yes | No | •fragrance •preservative •food additive Source***:*** https://pubchem.ncbi.nlm.nih.gov/compound/8456#section=Uses | 25013-16-5 | 89% | 40.7 | 6.6 | 118.0 | 31.6 | <1,000,000 lbs |
| **Citral A** | consumer products, personal care | Yes | Yes | No | • found in household cleaning products  • food additive  • fragrance  Source: https://pubchem.ncbi.nlm.nih.gov/compound/638011#section=Uses | 5392-40-5 | 85% | 33.1 | 5.9 | 97.7 | 30.5 | 100,000 - 500,000 lb |
| **B-citronellol** | personal care, consumer products | Yes | Yes | Yes | •food flavoring agent  •fragrance  •used in pesticides •found in household cleaning products, cosmetics, colognes, perfumes Source: https://pubchem.ncbi.nlm.nih.gov/compound/8842#section=Uses | 106-22-9 | 81% | 45.3 | 8.3 | 180.0 | 36.9 | 1,000,000 - 10,000,000 lb |
| **N,N-Diethyl-m-toluamide** | pesticides | No | No | Yes | • aka DEET, insect repellant  • pesticide Source: https://pubchem.ncbi.nlm.nih.gov/compound/4284#section=Uses | 134-62-3 | 81% | 921.2 | 18.6 | 9770.0 | 240.0 | **-** |
| **Di-n-nonyl phthalate** | chemicals in commerce | No | No | No | • used for making vinyl mixes to withstand heat  • plasticizer for vinyl resins  • used in industrial manufacturing  • found in toys  Source: https://pubchem.ncbi.nlm.nih.gov/compound/6787#section=Uses | 84-76-4 | 67% | 354.1 | 28.9 | 1370.0 | 177.5 | 100,000 - 500,000 lb |
| **Coumarin** | consumer products, personal care | Yes | Yes | Yes | • used in air fresheners  • used in the maintenance and repair of automobiles  • used in cleaning products  • found in pharmaceuticals  • food additive  • fragrance  • human metabolite  • related to manufacturing  • found in personal care products  • found in pesticides  Source: https://pubchem.ncbi.nlm.nih.gov/compound/323#section=Uses | 91-64-5 | 56% | 61.8 | 6.9 | 270.0 | 21.5 | 500,000 - 1,000,000 lb |
| **Diethyl phthalate** | chemicals in commerce, pesticides | Yes | Yes | Yes | • plasticizer  • vehicle for fragrances and cosmetic ingredients  • solvent  • found in air fresheners  • found in agents of chemical warfare  • used in cleaning products  • colorant  • inactive ingredient in pharmaceuticals  • found in food service and production facilities  • fragrance • found in personal care products  • used in pesticides  • found in toys  Source: https://pubchem.ncbi.nlm.nih.gov/compound/6781#section=Uses | 84-66-2 | 52% | 1216.5 | 28.3 | 4790.0 | 509.5 | 1,000,000 - 10,000,000 lb |
| **Tributyl phosphate** | chemicals in commerce, flame retardant | No | No | Yes | • flame retardant in aircraft hydraulic fluid  • solvent  • used in the making of plastics and in cement casings for oil wells  • used in building materials  • inert ingredient in pesticides  • softener for plastics  • used in toys  Source: https://pubchem.ncbi.nlm.nih.gov/compound/31357#section=Uses | 126-73-8 | 44% | 56.9 | 13.2 | 240.0 | 41.2 | 1,000,000 - 10,000,000 lb |
| **Benzothiazole** | chemicals in commerce | Yes | Yes | No | • colorant • food additive  • fragrance  Source: https://pubchem.ncbi.nlm.nih.gov/compound/7222#section=Uses | 95-16-9 | 44% | 9.8 | 3.2 | 16.1 | 8.5 | 1,000,000 - 10,000,000 lb |
| **Cinnamal** | chemicals in commerce, consumer products, personal care, pesticides | Yes | Yes | Yes | • fragrance • food additive  • found in pesticides  Source: https://pubchem.ncbi.nlm.nih.gov/compound/637511#section=Uses | 104-55-2 | 41% | 13.2 | 2.5 | 87.9 | 6.4 | 1,000,000 - 20,000,000 lb |
| **Dimethyl phthalate** | chemicals in commerce, pesticides | No | No | Yes | • increases flexibility of plastics and rubber  • used in rocket fuel, lacquers, and coating agents  • anti-caking agent  • used in building construction  • inactive ingredient in pharmaceuticals  • used to be in pesticides, but not any more  Source: https://pubchem.ncbi.nlm.nih.gov/compound/8554#section=Uses | 131-11-3 | 37% | 56.8 | 10.1 | 223.0 | 29.2 | 1,000,000 - 10,000,000 lb |
| **Permethrin** | pesticides | No | No | Yes | •mosquito control  •wood preservative, used on food/feed crops, farm animals, barns •used for head lice and scabies treatment  Source***:*** https://pubchem.ncbi.nlm.nih.gov/compound/40326#section=Overview | 52645-53-1 | 37% | 2975.8 | 8.0 | 25500.0 | 45.2 | **-** |
| **Caffeine** | consumer products, pharmacological | No | Yes | Yes | • used in cigarettes  • used in pharmaceuticals  • food additive  • human metabolite  • found in personal care products  • found in pesticides  Source: https://pubchem.ncbi.nlm.nih.gov/compound/2519#section=Uses | 58-08-2 | 37% | 158.6 | 28.3 | 345.0 | 175.0 | 100,000 - 500,000 lb |
| **Linalool** | personal care, pesticides | Yes | Yes | Yes | • food additive  • fragrance (floral, spicy, wood odor) • used in household cleaning products  • used in pesticide  • found in cigarettes  Source: https://pubchem.ncbi.nlm.nih.gov/compound/6549#section=Uses | 78-70-6 | 37% | 30.1 | 5.5 | 90.0 | 23.0 | 1,000,000 - 10,000,000 lb |
| **Ethofenprox** | pesticides | No | No | Yes | • pesticides Source: https://pubchem.ncbi.nlm.nih.gov/compound/71245#section=Uses | 80844-07-1 | 33% | 180.4 | 16.0 | 764.0 | 40.5 | **-** |
| **Bifenthrin** | pesticides | No | No | Yes | • insecticide Source: https://pubchem.ncbi.nlm.nih.gov/compound/6442842#section=Uses | 82657-04-3 | 26% | 500.1 | 10.4 | 2010.0 | 71.7 | - |
| **Quinoline** | chemicals in commerce | Yes | Yes | No | • used in cigarettes  • food additive  • fragrance • related to growing flowers or fruit  Source: https://pubchem.ncbi.nlm.nih.gov/compound/7047#section=Uses | 91-22-5 | 26% | 7.8 | 1.8 | 14.7 | 9.3 | <1,000,000 lbs |
| **Anthracene** | polycyclic aromatic hydrocarbons (PAHs) | Yes | No | Yes | • used to make dyes  • fragrance • pesticide  • polyaromatic hydrocarbons (PAHs) • used in surface treatment for inhibition of corrosion, rust, water  • used in automotive industry  • occurs as a result of incomplete burning  Source: https://pubchem.ncbi.nlm.nih.gov/compound/8418#section=Overview | 120-12-7 | 22% | 3.8 | 1.2 | 10.7 | 2.6 | 25,000 - 100,000 lb |
| **Triethyl phosphate** | chemicals in commerce, flame retardant, pesticides | No | No | Yes | • solvent • plasticizer for resins, plastics, and gums  • catalyst in the production of acetic anhydride by the ketene process  • fire retardant  • used in building construction  • used in chemical warfare  • inert ingredient in pesticides  Source: https://pubchem.ncbi.nlm.nih.gov/compound/6535#section=Uses | 78-40-0 | 22% | 34.4 | 4.3 | 132.0 | 18.8 | 1,000,000 - 10,000,000 lb |
| **Lyral** | personal care | Yes | No | Yes | • used in cleaning products  • fragrance  • used in personal care products  • inert ingredient in pesticides  Source: https://pubchem.ncbi.nlm.nih.gov/compound/91604#section=Uses | 31906-04-4 | 19% | 23.7 | 15.7 | 31.9 | 26.3 | <1,000,000 lbs |
| **4-Tert-butylphenol** | chemicals in commerce, consumer products | No | No | No | •intermediate in varnish and lacquer resin manufacturing •plasticizer Source: https://drugs.ncats.io/substance/O81VMW36CV; https://pubchem.ncbi.nlm.nih.gov/compound/7393#section=Uses | 98-54-4 | 19% | 26.9 | 7.0 | 75.0 | 16.4 | 10,000,000 - 50,000,000 lb |
| **Dicyclohexyl phthalate** | chemicals in commerce | No | No | No | • increases the flexibility of plastics  • used in paper finishes to make printer ink water resistant  • used in building materials  Source: https://pubchem.ncbi.nlm.nih.gov/compound/6777#section=Uses | 84-61-7 | 15% | 244.1 | 11.5 | 840.0 | 62.4 | 500,000 - 1,000,000 lb |
| **1-Methylnaphthalene** | chemicals in commerce, polycyclic aromatic hydrocarbons (PAHs), volatile organic compounds (VOCs) | Yes | Yes | Yes | • used in animal husbandry • pesticide  • food colorant • anti foaming agent • food additive • fragrance  Source: https://pubchem.ncbi.nlm.nih.gov/compound/7002#section=Uses | 90-12-0 | 15% | 0.9 | 0.5 | 1.3 | 0.9 | 1,000,000 - 10,000,000 lb |
| **2,4-Di-tert-butylphenol** | chemicals in commerce | No | No | No | • maintenance and repair of automobiles • lubricant for engines, brake fluids, oil • plastics • personal care products • in solvents  Source: https://pubchem.ncbi.nlm.nih.gov/compound/7311#section=Uses | 96-76-4 | 15% | 21.1 | 9.2 | 36.2 | 19.5 | 10,000,000 - 50,000,000 lb |
| **Permethrin II** | pesticides | No | No | Yes | • pesticide | 999046-03-6 | 15% | 9091.1 | 89.5 | 33000.0 | 1637.5 | **-** |
| **Benzyl alcohol** | chemicals in commerce, consumer products, personal care, pharmacological | Yes | Yes | Yes | • used as a solvent • used as a preservative  • fragrance  • used in personal care products • colorant • used in air fresheners  • used in cigarettes  • found in cleaning products  • found in pharmaceuticals • found in electronics  • food additive  • found in pesticides  • used in rubber products and manufacturing  Source: https://pubchem.ncbi.nlm.nih.gov/compound/244#section=Uses | 100-51-6 | 11% | 52.4 | 8.9 | 126.0 | 22.3 | 10,000,000 - 50,000,000 lb |
| **Benzyl cinnamate** | consumer products, personal care | Yes | Yes | No | • food additive  • fragrance  Source: https://pubchem.ncbi.nlm.nih.gov/compound/5273469#section=Use-and-Manufacturing | 103-41-3 | 11% | 136.3 | 2.8 | 250.0 | 156.0 |  |
| **Hydroxy-citronellal** | personal care | Yes | Yes | Yes | • fragrance  • used in air fresheners  • used in cigarettes  • used in cleaning products  • food additive  • used in personal care products  • inert ingredient in pesticides  Source: https://pubchem.ncbi.nlm.nih.gov/compound/7888#section=Uses | 107-75-5 | 11% | 185.5 | 48.5 | 386.0 | 122.0 | <1,000,000 lbs |
| **PBDE 49** | flame retardant | No | No | No | No specific uses  Sources: https://pubchem.ncbi.nlm.nih.gov/compound/15509892 | 243982-82-3 | 11% | 25.1 | 6.2 | 47.0 | 22.0 | **-** |
| **Piperonyl butoxide** | pesticides | No | Yes | Yes | • pesticides  • food additive  • used in personal care products  Source: https://pubchem.ncbi.nlm.nih.gov/compound/5794#section=Uses | 51-03-6 | 11% | 1068.0 | 764.0 | 1300.0 | 1140.0 | 500,000 - 1,000,000 lb |
| **Cypermethrin-2** | pesticides | No | No | Yes | • pesticide Source: https://pubchem.ncbi.nlm.nih.gov/compound/2912#section=Uses | 52315-07-8 | 11% | 63.6 | 21.7 | 89.1 | 80.0 | **-** |
| **Tri-p-tolyl phosphate** | chemicals in commerce | No | No | Yes | • used in flexible PVC and rubbers  • used in blends with phthalates  • used in building materials  • flame retardant  • used as a preservative in pesticides  Source: https://pubchem.ncbi.nlm.nih.gov/compound/6529#section=Uses | 78-32-0 | 11% | 23.5 | 11.7 | 39.6 | 19.1 | 5,000,000,000 - 10,000,000,000 lb |
| **Tris(2-ethylhexyl) phosphate** | chemicals in commerce, flame retardant | No | No | Yes | • used as a solvent in the production of hydrogen peroxide  • used as a vinyl plasticizer with flame retardant properties • used in building construction  • inert ingredient in pesticides  Source: https://pubchem.ncbi.nlm.nih.gov/compound/6537#section=Uses | 78-42-2 | 11% | 175.3 | 140.0 | 210.0 | 176.0 | 1,000,000 - 20,000,000 lb |
| **Musk Ketone** | personal care | Yes | Yes | No | • fragrance (sweet, persistent odor) • used in household cleaning products  • food additive  • used in personal care products  Source: https://pubchem.ncbi.nlm.nih.gov/compound/6669#section=Uses | 81-14-1 | 11% | 140.0 | 24.2 | 332.0 | 63.9 | < 25,000 lb |
| **Di-n-hexyl phthalate** | chemicals in commerce | No | No | Yes | • inert ingredient in pesticides  • found in food packaging  • found in plastics and rubber  Source: https://pubchem.ncbi.nlm.nih.gov/compound/6786#section=Use-and-Manufacturing | 84-75-3 | 11% | 377.6 | 41.1 | 1030.0 | 61.8 | 100,000 - 500,000 lb |
| **4-Isopropylphenol** | chemicals in commerce, personal care | **No** | **Yes** | **Yes** | • food additive  • related to crude oil and oil products  • inert ingredient in pesticides Source: https://pubchem.ncbi.nlm.nih.gov/compound/7465#section=Use-and-Manufacturing | 99-89-8 | 11% | 4.3 | 3.8 | 4.9 | 4.3 | <1,000,000 lbs |
| **TCEP** | flame retardant | Yes | No | Yes | • used in rigid foams  • used in flame retardant coatings  • used in building materials  • fragrance  • used in pesticides  Source: https://pubchem.ncbi.nlm.nih.gov/compound/8295#section=Uses | 115-96-8 | 7% | 105.6 | 83.1 | 128.0 | 105.6 | 25,000 - 100,000 lb |
| **Di-n-octyl phthalate** | chemicals in commerce | No | No | Yes | • added to plastics to make them flexible  • used in building materials  • found in electronics  • found in food packaging  • found in pesticides  • found in toys Source: https://pubchem.ncbi.nlm.nih.gov/compound/8346#section=Uses | 117-84-0 | 7% | 919.3 | 58.6 | 1780.0 | 919.3 | **-** |
| **A-Ionone** | personal care | Yes | Yes | Yes | • used as an absorbent  • found in cigarettes  • found in cleaning products, including soaps and detergents • found in flooring materials  • food additive  • food flavoring  • fragrance  • found in beverages for human consumption  • found in food for human consumption  • found in personal care products  • found in pesticides  Source: https://pubchem.ncbi.nlm.nih.gov/compound/5282108#section=Uses | 127-41-3 | 7% | 153.2 | 21.4 | 285.0 | 153.2 | 100,000 - 500,000 lb |
| **2-Methylphenanthrene** | polycyclic aromatic hydrocarbons (PAHs) | No | No | No | No specific uses   Source: https://pubchem.ncbi.nlm.nih.gov/compound/17321 | 2531-84-2 | 7% | 3.7 | 2.1 | 5.4 | 3.7 | **-** |
| **Cashmeran** | personal care | Yes | No | No | • used in home cleaning products  • fragrance  • found in personal care products  Source: https://pubchem.ncbi.nlm.nih.gov/compound/92292#section=Uses | 33704-61-9 | 7% | 32.9 | 30.3 | 35.5 | 32.9 | <1,000,000 lbs |
| **Hydroprene** | pesticides | No | No | Yes | • used in pesticides  Source: https://pubchem.ncbi.nlm.nih.gov/compound/5372477#section=Use-and-Manufacturing | 41096-46-2 | 7% | 237.5 | 115.0 | 360.0 | 237.5 | **-** |
| **Farnesol I** | consumer products, personal care | Yes | Yes | Yes | • food additive  • used in pesticides  • fragrance  Source: https://pubchem.ncbi.nlm.nih.gov/compound/445070#section=Use-and-Manufacturing | 4602-84-0 | 7% | 31.8 | 25.4 | 38.1 | 31.8 | **-** |
| **PBDE 47** | flame retardant | No | No | No | • flame retardant  Source: https://pubchem.ncbi.nlm.nih.gov/compound/95170#section=Use-and-Manufacturing | 5436-43-1 | 7% | 32.9 | 18.1 | 47.7 | 32.9 | **-** |
| **Tricresylphosphate, meta-** | chemicals in commerce, flame retardant | No | No | No | • plasticizer in vinyl plastics  • flame retardant • solvent  Source: https://pubchem.ncbi.nlm.nih.gov/compound/11232#section=Use-and-Manufacturing | 563-04-2 | 7% | 28.7 | 25.9 | 31.5 | 28.7 | **-** |
| **D-Limonene** | consumer products, personal care | Yes | Yes | Yes | • fragrance • used as a solvent  • pesticide  • used in air fresheners  • used in automotive care  • found in building materials  • found in cleaning products  • colorant • found in electronics  • food additive  • used in manufacturing  • found in personal care products  Source: https://pubchem.ncbi.nlm.nih.gov/compound/440917#section=Uses | 5989-27-5 | 7% | 66.5 | 63.0 | 70.0 | 66.5 | 1,000,000 - 10,000,000 lb |
| **Phthalimide** | chemicals in commerce, pesticides | No | No | Yes | • inert ingredient in pesticides  Source: https://pubchem.ncbi.nlm.nih.gov/compound/6809#section=Use-and-Manufacturing | 85-41-6 | 7% | 33.2 | 18.1 | 48.3 | 33.2 | 25,000 - 100,000 lb |
| **4-Chloro-3,5-dimethylphenol** | chemicals in commerce | **Yes** | **Yes** | **Yes** | • pesticide • used in cleaning products  • colorant for food or personal care products • found in pharmaceuticals  • found in food packaging • fragrance  • found in personal care products  Source: https://pubchem.ncbi.nlm.nih.gov/compound/2723#section=Uses | 88-04-0 | 7% | 10.5 | 6.0 | 15.0 | 10.5 | <1,000,000 lbs |
| **Naphthalene** | polycyclic aromatic hydrocarbons (PAHs), volatile organic compounds (VOCs) | Yes | Yes | Yes | • also called white tar and tar camphor  • used in the manufacture of chemicals used as softeners in PVC plastics  • used as a moth repellant and toilet deodorant blocks • burning wood, tobacco, or fossil fuels releases it, as fuels naturally contain it  • pesticide  • used in fragrance  • food additive for flavor  Source: https://pubchem.ncbi.nlm.nih.gov/compound/931#section=Uses | 91-20-3 | 7% | 0.7 | 0.4 | 1.0 | 0.7 | 100,000,000 - 250,000,000 lb |
| **Eugenol** | chemicals in commerce, consumer products, personal care, pesticides, pharmalogical | Yes | Yes | Yes | • fragrance, clove and carnation  • used in cleaning products  • used in pharmaceutical products • food additive  • used in pesticides • found in personal care products  Source: https://pubchem.ncbi.nlm.nih.gov/compound/3314#section=Uses | 97-53-0 | 7% | 6.7 | 6.5 | 6.9 | 6.7 | 1,000,000 - 10,000,000 lb |
| **Geraniol** | consumer products, personal care | Yes | Yes | Yes | • fragrance • used in pesticides • used in air fresheners  • used in cigarettes • used in cleaning products  • food additive  • used in personal care products  Source: https://pubchem.ncbi.nlm.nih.gov/compound/637566#section=Uses | 106-24-1 | 4% | 41.0 | 41.0 | 41.0 | 41.0 | 1,000,000 - 10,000,000 lb |
| **Isobornyl thiocyanoacetate** | pesticides | No | No | Yes | • pesticide, although not used anymore  Source: https://pubchem.ncbi.nlm.nih.gov/compound/8267#section=Use-and-Manufacturing | 115-31-1 | 4% | 1470.0 | 1470.0 | 1470.0 | 1470.0 | **-** |
| **2,6-Di-tert-butylphenol** | chemicals in commerce | **Yes** | **No** | **No** | • used in agriculture • used in construction  • fragrance • used in food production  • lubricant for engines, brake fluids, oil • related to crude oil and oil products  • related to paper manufacturing  • plastics • personal care products  • used in rubber products and manufacturing  • stabilizer  • treatments for inhibiting rust, corrosion, water Source: https://pubchem.ncbi.nlm.nih.gov/compound/31405#section=Uses | 128-39-2 | 4% | 3.5 | 3.5 | 3.5 | 3.5 | 100,000,000 - 250,000,000 lb |
| **Pyrene** | chemicals in commerce, polycyclic aromatic hydrocarbons (PAHs) | No | No | Yes | • polycyclic aromatic hydrocarbons (PAHs) • occurs as a result of incomplete burning • found in tobacco smoke, automobile exhaust, griller or smoked meat or fish  • used in pesticides • used in personal care products  Source: https://pubchem.ncbi.nlm.nih.gov/compound/31423#section=Uses | 129-00-0 | 4% | 2.1 | 2.1 | 2.1 | 2.1 | 100,000 - 500,000 lb |
| **Celestolide** | consumer products, personal care | Yes | Yes | No | • used as a fragrance additive in soaps and cosmetics  • food additive  Source: https://pubchem.ncbi.nlm.nih.gov/compound/61585#section=Uses | 13171-00-1 | 4% | 4.2 | 4.2 | 4.2 | 4.2 | **-** |
| **Acenaphthylene** | polycyclic aromatic hydrocarbons (PAHs) | Yes | No | No | • used in candles  • fragrance • polyaromatic hydrocarbons (PAHs)  Source: https://pubchem.ncbi.nlm.nih.gov/compound/9161#section=Uses | 208-96-8 | 4% | 2.8 | 2.8 | 2.8 | 2.8 | **-** |
| **D-(cis-trans)-Phenothrin-I** | pesticides | No | No | Yes | • pesticide Source: https://pubchem.ncbi.nlm.nih.gov/compound/4767#section=Uses | 26002-80-2 | 4% | 83.1 | 83.1 | 83.1 | 83.1 | **-** |
| **Promecarb** | pesticides | No | No | Yes | • pesticide Source: https://pubchem.ncbi.nlm.nih.gov/compound/17516#section=Use-and-Manufacturing | 2631-37-0 | 4% | 23.0 | 23.0 | 23.0 | 23.0 | **-** |
| **Triclosan** | chemicals in commerce, personal care, pharmacological | Yes | No | Yes | • used as a bacteriocide in cosmetics and personal care products  • used in cleaning products  • used in pharmaceuticals  • used in food service activities  • fragrance  • used in pesticides  • found in toys  Source: https://pubchem.ncbi.nlm.nih.gov/compound/5564#section=Uses | 3380-34-5 | 4% | 11.9 | 11.9 | 11.9 | 11.9 | **-** |
| **Tilt** | pesticides | No | Yes | Yes | • pesticide • food additive  • flame retardant  • used in manufacturing of building material  Source: https://pubchem.ncbi.nlm.nih.gov/compound/43234#section=Uses | 60207-90-1 | 4% | 137.0 | 137.0 | 137.0 | 137.0 | **-** |
| **Buprofezin** | pesticides | No | No | Yes | • pesticide  Source: https://pubchem.ncbi.nlm.nih.gov/compound/50367#section=Uses | 69327-76-0 | 4% | 45.7 | 45.7 | 45.7 | 45.7 | - |
| **Phenanthrene** | polycyclic aromatic hydrocarbons (PAHs) | Yes | No | Yes | • polycyclic aromatic hydrocarbons (PAHs) • occurs in cigarette smoke, gasoline and diesel engine exhaust  • used in fertilizers  • fragrance  • used in pesticides  Source: https://pubchem.ncbi.nlm.nih.gov/compound/995#section=Uses | 85-01-8 | 4% | 3.4 | 3.4 | 3.4 | 3.4 | 500,000 - 1,000,000 lb |
| **Thymol** | pharmacological | Yes | Yes | Yes | • constituent of oil of thyme, which naturally occurs in thyme  • used in cigarettes  • used in cleaning products  • used in pharmaceuticals  • food additive  • fragrance  • human metabolite  • occurs in personal care products  • used in pesticides  Source: https://pubchem.ncbi.nlm.nih.gov/compound/6989#section=Uses | 89-83-8 | 4% | 24.0 | 24.0 | 24.0 | 24.0 | <1,000,000 lbs |
| **Methyleugenol** | personal care | Yes | Yes | Yes | • food additive  • found in fragrance  • found in personal care products  • found in pesticides  Source: https://pubchem.ncbi.nlm.nih.gov/compound/7127#section=Uses | 93-15-2 | 4% | 16.6 | 16.6 | 16.6 | 16.6 | **-** |
| **2,4-Dimethylaniline** | chemicals in commerce | No | Yes | No | • colorant for food or personal care products • related to paper manufacturing Source: https://pubchem.ncbi.nlm.nih.gov/compound/7250#section=Use-and-Manufacturing | 95-68-1 | 4% | 11.9 | 11.9 | 11.9 | 11.9 | <1,000,000 lbs |
| **Carvone** | consumer products, personal care, pesticides | Yes | Yes | Yes | • used as flavoring agent  • found in personal care products, including toothpaste • used in cigarettes  • food additive  • found in pesticides  Source: https://pubchem.ncbi.nlm.nih.gov/compound/7439#section=Uses | 99-49-0 | 4% | 14.4 | 14.4 | 14.4 | 14.4 | **-** |
|  |  |  |  |  |  |  |  |  |  |  |  |  |
|  |  |  |  |  |  |  |  |  |  |  |  |  |
| Note: Chemicals with no production volume data were not present in the EPA Chemical Data Reporting database, which can be accessed here: https://www.epa.gov/chemical-data-reporting/access-cdr-data#2016 | | | | | |  |  |  |  |  |  |  |
